# Reliability of Large Language Model Generated Clinical Reasoning in Assisted Reproductive Technology: Blinded Comparative Evaluation Study

**DOI:** 10.2196/85206

**Published:** 2026-01-08

**Authors:** Dou Liu, Ying Long, Sophia Zuoqiu, Di Liu, Kang Li, Yiting Lin, Hanyi Liu, Rong Yin, Tian Tang

**Affiliations:** 1 Department of Obstetrics and Gynecology West China Second University Hospital of Sichuan University Chengdu China; 2 Department of Industrial and Operations Engineering University of Michigan Ann Arbor, MI United States; 3 Key Laboratory of Birth Defects and Related Diseases of Women and Children Sichuan University Chengdu China; 4 Reproductive Medical Center, Department of Obstetrics and Gynecology West China Second University Hospital of Sichuan University Chengdu China; 5 Department of Industrial Engineering Sichuan University Chengdu China; 6 West China Biomedical Big Data Center West China Hospital of Sichuan University Chengdu China; 7 Med-X Center for Informatics Sichuan University Chengdu China; 8 West China School of Medicine Sichuan University Chengdu China

**Keywords:** chain-of-thought, large language model, assisted reproductive technology, explainable artificial intelligence, clinical data reliability

## Abstract

**Background:**

High-quality clinical chains-of-thought (CoTs) are essential for explainable medical artificial intelligence (AI); yet, their development is limited by data scarcity. Large language models can generate medical CoTs, but their clinical reliability is unclear.

**Objective:**

We evaluated the clinical reliability of large language model–generated CoTs in reproductive medicine and examined prompting strategies to improve their quality.

**Methods:**

In a blinded comparative study at a clinical center, senior clinicians in assisted reproductive technology evaluated CoTs generated via 3 distinct strategies: zero-shot, random few-shot (using random shallow examples), and selective few-shot (using diverse, high-quality examples). Expert ratings were then compared with evaluations from a state-of-the-art AI model (GPT-4o).

**Results:**

The selective few-shot strategy significantly outperformed other strategies across logical clarity, use of key information, and clinical accuracy (*P*<.001). Critically, the random few-shot strategy offered no significant improvement over the zero-shot baseline, demonstrating that low-quality examples are as ineffective as no examples. The success of the selective strategy is attributed to 2 preliminary frameworks: “gold-standard depth” and “representative diversity.” Notably, the AI evaluator failed to discern these critical performance differences. Thus, clinical reliability depends on strategic prompt design rather than simply adding examples.

**Conclusions:**

We propose a “dual principles” preliminary framework for generating trustworthy CoTs at scale in assisted reproductive technology. This work is a preliminary step toward addressing the data bottleneck in reproductive medicine. It also underscores the essential role of human expertise in evaluating generated clinical data.

## Introduction

### Background

Assisted reproductive technology (ART) represents a cornerstone of modern medicine, offering solutions for millions facing infertility [1]. The clinical decision-making process in ART is exceptionally complex, requiring the synthesis of high-dimensional patient data, including baseline characteristics and medical history. This process is time-consuming and fraught with risk for both clinicians and patients, as minute variations in treatment protocols can lead to significant adverse outcomes. Furthermore, clinicians must navigate patients’ personal values and ethical considerations, demanding a highly personalized and explainable approach to care [2].

Recent advancements in artificial intelligence (AI), particularly large language models (LLMs), have demonstrated considerable promise for answering medical questions, addressing clinical case challenges, and augmenting clinical diagnosis [3-7]. Within clinical decision support systems, these technologies can help synthesize large amounts of data, facilitating more comprehensive and standardized therapeutic strategies. However, while general-purpose LLMs like ChatGPT-4 and Gemini are powerful, their training on broad, nonspecialized data limits their utility in niche medical domains. Consequently, high-performing clinical AI applications are typically fine-tuned from general models using curated, domain-specific datasets [8-10]. The actual bottleneck, however, is not a lack of raw clinical data, but a profound lack of explainable data—data that record not just what decision was made, but why. This meticulous, expert-level reasoning, often captured as a chain-of-thought (CoT), is the very fuel required to train AI models that are not just accurate, but also trustworthy and scalable to clinicians. To move beyond “black-box” predictions, models require structured reasoning pathways, or CoT data, which simulate clinical logic and enhance explainability [11,12]. The challenge, therefore, narrows down to a scarcity of expert-authored CoT data within the specific area. The manual creation of such a dataset on a large scale is prohibitively expensive and time-consuming, presenting a significant barrier to progress in explainable medical AI.

To address this challenge, a promising direction is to leverage the generative capabilities of LLMs to synthesize clinical CoT data at scale. While this offers a scalable solution to the data bottleneck, it hinges on a critical, unverified assumption: the clinical reliability of the generated content. In a high-stakes domain like ART, this assumption cannot be taken for granted.

**Figure 1:**
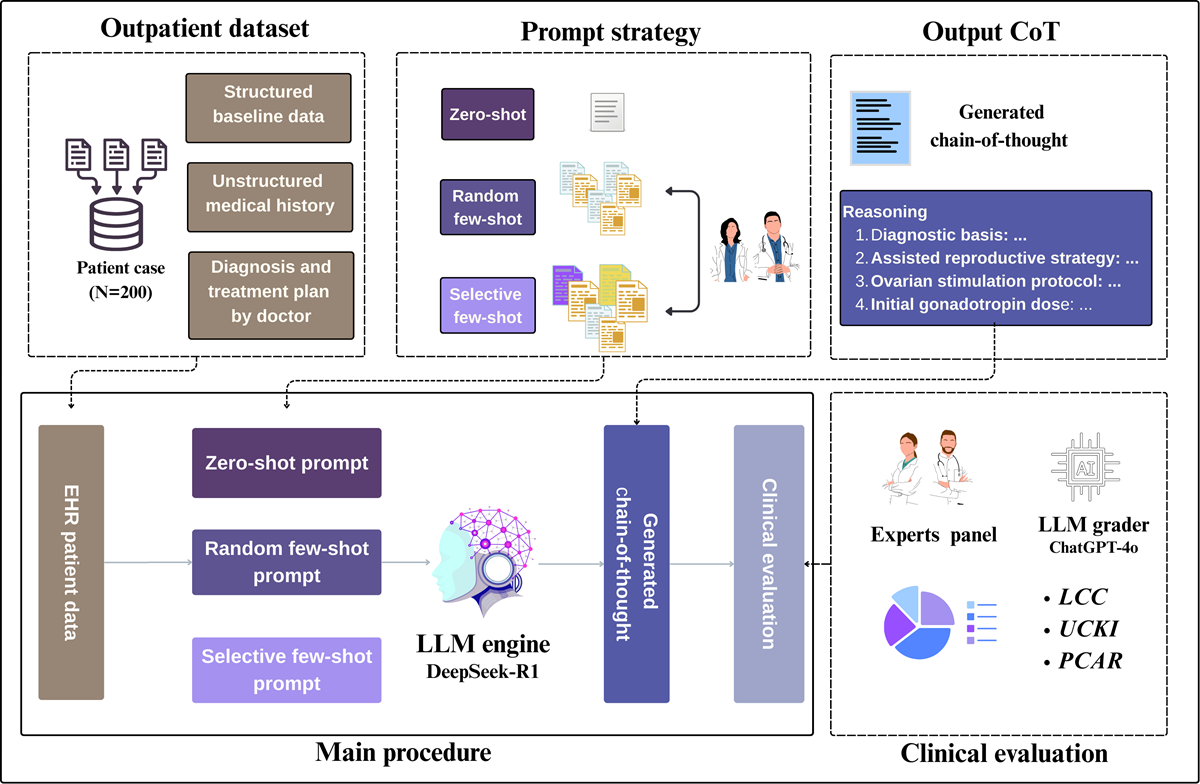
Conceptual framework of the comparative evaluation study. EHR: electronic health record; LCC: logical coherence and clarity; LLM: large language model; PCAR: plausibility and clinical accuracy of reasoning; UCKI: use and coverage of key information.

Therefore, this study is designed to examine this uncertainty under the ART setting through a rigorous, head-to-head empirical comparison. Figure 1 presents the conceptual framework of our comparative evaluation study. We hypothesize that a selective few-shot strategy, meticulously crafted with diverse and deeply reasoned examples, will improve the factual accuracy, logical clarity, and clinical information use of LLM-generated reasoning in infertility decision-making scenarios, compared with generic zero-shot and random few-shot prompting. To test this, we developed a novel prompting framework and validated it through a blinded evaluation protocol where senior clinicians from the reproductive department assessed the quality of CoTs from all 3 strategies. In a secondary analysis, we further contrast these expert assessments against the state-of-the-art (SOTA) AI evaluator (GPT-4o) at that time to critically examine the current capabilities and limitations of automated evaluation paradigms, which are widely used in supervised data generation. Ultimately, this work aims to establish an exploratory, practical, evidence-based methodology for the trustworthy generation of clinical reasoning of ART at scale.

The study workflow begins with a standardized patient case (N=200) as the input. Three distinct prompting strategies are evaluated in parallel pipelines: (1) zero-shot, which uses instructions only; (2) random few-shot, which uses 5 randomly selected examples; and (3) selective few-shot, which uses a curated set of 6 diverse examples representing all major ART categories in the cases. Each strategy, powered by the same LLM engine, generates a unique CoT. All 3 generated CoTs are then subjected to a rigorous, blinded “Doctor-in-the-Loop” evaluation by 2 parallel assessors: human clinical experts (the gold standard) and the SOTA AI evaluator (GPT-4o). This dual evaluation process yields the final reliability scores and rankings for each strategy.

### LLMs in Health Care

Since the launch of ChatGPT-4, LLMs have rapidly spread into many industries, such as education, finance, and health care. For instance, Google’s Med-PaLM 2, a leading specialized health care model, achieved 86.5% accuracy on the MedQA benchmark, a popular multiple-choice open domain question answering (OpenQA) medical problems dataset. Furthermore, its responses were preferred over those of generalist physicians in 65% of expert evaluations [13]. The LLMs are now used in many healthcare–related workflows, ranging from medical documentation assistance to clinical differential diagnosis [5,14-16]. However, to effectively address highly specialized tasks, these models are typically fine-tuned from pretrained LLMs using carefully curated datasets. Despite their impressive capabilities, current LLMs usually function as black boxes, producing outputs without offering interpretable reasoning. In clinical practice, however, physicians often require not just answers but also transparent explanations. This requires models beyond black-box behavior and provides interpretable, step-by-step reasoning processes. The increasing reliance on LLMs has also intensified the demand for high-quality data [17]. Alarmingly, some predict that the global supply of novel text data may be exhausted by 2050 and image data by 2060 [18]. In the health care domain, the situation is even more critical: clinical data are not only scarce but also highly sensitive and expensive to obtain. As a result, a central challenge emerges—how can we build datasets that are both sufficiently large and clinically trustworthy to support transparent, reliable medical AI systems?

### Synthetic Data in Health Care

To overcome the data shortage in health care, researchers are increasingly turning to LLMs to create synthetic data. This approach is promising for several reasons. It allows for data generation at scale, addressing issues of data scarcity and privacy [19,20]. Furthermore, synthetic data can be tailored to balance underrepresented patient groups, potentially improving model robustness and fairness [21]. Generative models have demonstrated remarkable success in these areas, with some studies showing that LLM-generated narratives can be indistinguishable from those written by physicians [22]. This potential, however, is inextricably linked to a profound challenge: reliability. While LLMs can mimic the style of clinical text, ensuring the factual accuracy and clinical plausibility of the content is a far more difficult task. For instance, models have been used to generate both structured electronic health records (EHRs) and unstructured clinical notes [23], but in both cases, the risk of hallucination—where the model generates incorrect or nonsensical information—poses a significant threat in high-stakes medical applications. Therefore, the core challenge moves beyond mere data generation to a more fundamental question of trust. While studies have shown that synthetic data can be effective for certain human labeling and fine-tuning tasks [24-28], these applications often involve relatively straightforward data points. The problem is magnified when the task requires complex, multistep medical reasoning. In such scenarios, “synthetic” must not equate to “inaccurate.” This underscores the urgent need for rigorous evaluation methods, not just for the data points themselves, but for the underlying reasoning processes that produce clinical decisions. Our work focuses on this critical next step: assessing the reliability of synthetically generated reasoning paths.

### Chain-of-Thought

CoT is a prompt engineering technique that enhances the output of LLMs, particularly for complex tasks involving multistep reasoning. It facilitates problem-solving by guiding the model through a step-by-step reasoning process by using a coherent series of logical steps [29]. This approach has been shown to significantly elevate performance on a wide range of complex reasoning tasks in general domains, especially for arithmetic problems and logical reasoning tasks [30,31]. To enhance the reasoning ability in domain-specific tasks, researchers have started fine-tuning the models with CoTs [12]. Within the medical domain, the potential of CoT is particularly compelling. Its step-by-step nature aligns naturally with the differential diagnosis and clinical reasoning processes used by physicians. Consequently, researchers have begun to apply CoT prompting to improve accuracy on medical question-answering benchmarks and in practice diagnosis [11,32]. More importantly, CoT offers a crucial pathway toward explainable AI in medicine. By externalizing the model’s reasoning processes, CoT allows clinicians to scrutinize, understand, and ultimately trust the AI’s recommendations, which is a prerequisite for its safe integration into clinical workflows.

The application of CoT is rapidly evolving. Beyond simple prompting, a new frontier in clinical AI is the (1) fine-tuning of models on datasets enriched with CoT data to build inherently more explainable systems, which, however, immediately confronts the fundamental bottleneck of medical AI; and the (2) prohibitive cost and time required for expert clinicians to manually author thousands of high-quality reasoning paths for a training set. An intuitive and scalable solution is to leverage foundational LLMs to synthetically generate these CoTs, creating a cost-effective pathway to train the next generation of trustworthy medical models. However, this entire paradigm hinges on a critical, yet largely unexamined, question: (3) Is the reliability of synthetically generated CoTs adequate to support their application in complex clinical scenarios? Literature to date offers little guidance. Most research focuses on the extrinsic value of CoT (ie, improving final answer accuracy), with scant attention paid to the intrinsic reliability of the reasoning itself. A model fine-tuned on flawed, albeit synthetically generated, logic could learn to produce seemingly correct answers for the wrong reasons—a risk that is unacceptable in clinical practice. Furthermore, standardized, expert-driven protocols for assessing the clinical validity, coherence, and faithfulness of machine-generated reasoning are conspicuously absent. Our study is designed to directly fill this foundational gap. Before the field can confidently use synthetic CoT for model training at scale, we must first have a rigorous method to measure its reliability. Therefore, we propose and implement a blinded, expert-led evaluation framework to answer the fundamental question: how reliable is synthetically generated sophisticated clinical reasoning, and what is the best prompting strategy to elicit it from LLMs?

## Methods

### Data Source

From the manually reviewed dataset, we randomly selected 200 cases as our evaluation set, covering a variety of ART. These ARTs are broadly categorized into 3 generations: in vitro fertilization (IVF), intracytoplasmic sperm injection (ICSI), and preimplantation genetic testing (PGT). Each generation includes several clinical subtypes, such as short-protocol IVF and IVF with donor sperm. Among the 200 evaluated cases, IVF accounts for the largest proportion (140/200, 70%), including standard IVF (116/200, 58%), IVF with donor sperm (9/200, 4.5%), and short-protocol IVF (short-time insemination, 15/200, 7.5%). The second most common is ICSI (38/200, 19%), comprising standard ICSI (26/200, 13%), IVF+ICSI (5/200, 2.5%), and TESA (testicular sperm aspiration)+ICSI (7/200, 3.5%). PGT represents 11% (22/200) of the dataset, including preimplantation genetic testing for aneuploidies (PGT-A; 6/200, 3%), preimplantation genetic testing for monogenic disorder (PGT-M; 3/200, 1.5%), and PGT for structural rearrangements (13/200, 6.5%).

As shown in Table 1, the dataset consisted of three main components: (1) a structured set of baseline and demographic variables, (2) the preliminary diagnosis and treatment plan, and (3) an unstructured narrative description of the present illness history. The structured baseline data served as the quantitative and categorical foundation for clinical assessment, encompassing key indicators of ovarian reserve such as anti-Müllerian hormone and baseline follicle-stimulating hormone levels. The unstructured narrative provided essential clinical context, offering a detailed account of the patient’s medical journey, which is critical for nuanced and context-aware medical reasoning. The output data, labeled as preliminary diagnosis and treatment plan, reflects the clinical conclusions and therapeutic strategies formulated by human experts in prior encounters. This component serves as the ground truth outcome. The LLM’s task is to generate a reasoning path that logically connects the patient’s input data to this expert-defined outcome. A detailed breakdown of all case data variables, including structured baseline indicators and narrative clinical history, is provided in Multimedia Appendix 1. Together, these inputs formed the foundation for CoT generation and model evaluation.

**Table 1:** Structure and description of input and output variables for each case^a^.

Category	Variable
Baseline and demographics	Female age Menstrual cycle Body weight BMI Anti-Müllerian hormone level Duration of infertility Gynecological ultrasound findings Baseline follicle-stimulating hormone level
Present illness history	Present illness history
Preliminary diagnosis and treatment plan	Type of infertility Controlled ovarian stimulation protocol Initial gonadotropin dosage Preliminary differential diagnosis Initial assisted reproductive technology strategy

^a^This table outlines the variables provided to the large language model for each case, categorized into input (baseline and demographic and present illness history) and output (preliminary diagnosis and treatment plan). These variables form the basis for the chain-of-thought generation task.

### Ethical Considerations

A set of EHRs recorded between 2020 and 2022 in the Infertility Outpatient Department at West China Second University Hospital was considered in this study. The study was approved by the institutional review board of West China Second University Hospital, Sichuan University (ID: 2022288). The EHRs have been manually reviewed and corrected to ensure data accuracy. All EHR data used in this study were fully deidentified before being accessed by the research team. The deidentification procedure followed HIPAA (Health Insurance Portability and Accountability Act) Safe Harbor standards, including the removal of all direct identifiers (eg, name, date of birth, medical record number, contact information, and provider information) and all quasi-identifiers (eg, dates, locations, and institutional identifiers). Only aggregated clinical descriptors necessary for the reasoning task (eg, high-level patient history and laboratory summaries) were retained. Access to the deidentified dataset was restricted to authorized study personnel through institution-managed credentials and encrypted storage. Reviewers performing the blinded evaluation accessed only the deidentified clinical vignettes and model-generated reasoning content through a secure, read-only interface; no downloads or reidentification attempts were permitted. All access was logged and monitored by an internal auditor to ensure compliance with institutional clinical data governance policies. Because the study used retrospective, fully deidentified EHRs, the requirement for updated informed consent for the following analysis was waived by the institutional review board in accordance with national regulations. No participants were contacted for this study, and no compensation was provided to participants.

### Experiment Design

#### Overview

To systematically evaluate the reliability of LLM-generated CoT and to determine the impact of different prompting strategies, we designed a comparative study. The experiment was structured into 3 distinct arms, each representing a different level of contextual information provided to the model. Our design philosophy was to create a controlled, stepwise comparison to isolate the effects of in-context examples and the strategy used for their selection.

All 3 groups used the evaluation dataset (N=200) described in the data source, making sure of a fair comparison. A capable “Teacher Model” is key to generating better-quality data [33]. Considering the models’ performance so far, we used the open-source model DeepSeek-R1-671b, which was known for its outstanding reasoning capability, as our consistent model shared by 3 arms [34]. Across all 3 arms, the LLM was assigned the same core task: generating a detailed, step-by-step ART CoT by integrating all provided patient information and the corresponding expert-provided reference output. All the inference was conducted via the application programming interface call. The model was executed with temperature=0.5 and max tokens=5000. We used temperature=0.5 to reflect typical clinical-LLM use, where deterministic decoding (temperature=0) may produce rigid or incomplete clinical reasoning. All prompting strategies were evaluated under identical generation settings to ensure fair comparison.

#### Group 1: Zero-Shot Baseline

In this group, we aimed to establish a fundamental baseline to evaluate the out-of-the-box clinical reasoning capabilities of general-purpose LLMs when applied to this specialized task. To this end, the model was prompted using a standardized directive instruction, with each clinical case embedded directly into the prompt (see Multimedia Appendix 2 for details). The outputs generated by the model, along with corresponding physician evaluations, served as a performance floor, quantifying the baseline reliability and limitations of an unadapted LLM in handling novel clinical scenarios.

#### Group 2: Random Few-Shot Prompting

This experimental arm was designed to establish a baseline for a standard, nonoptimized few-shot approach. Its purpose was to measure the impact of providing generic, in-domain examples without a specific selection strategy. For each of the 200 test cases, the prompt was initially prepared with a fixed set of 5 examples to provide context for the model. These 5 examples were randomly sampled from our expert-authored data pool, excluding the existing evaluation dataset. The sample set used in the prompt for every test case consisted of 4 standard IVF cases and 1 short-protocol IVF case, accompanied by a concise reasoning chain authored by domain experts. The prompt structure and instructions were otherwise identical to those in the other arms. A representative example of a few-shot sample, detailing the input data and expert-written CoT, is provided in Multimedia Appendix 2. This approach represents a “naive” few-shot implementation. It is designed to test the hypothesis that the mere presence of in-domain examples, even without being specifically tailored to the test case, is sufficient to improve reasoning quality compared to the zero-shot baseline.

#### Group 3: Selective Few-Shot Prompting

This arm represents our proposed method and was designed to test the hypothesis that a deliberately curated set of diverse examples would improve reasoning reliability and generalization. Instead of random sampling, this approach used a clinically informed, representative selection strategy. Physicians curated a set of 6 exemplary cases from a pool of records not included in the 200 evaluation set (to prevent data leakage). These 6 examples were specifically chosen to represent the full spectrum of major ART categories present in our dataset, including IVF (standard IVF, short-protocol IVF, and IVF with donor sperm), ICSI, TESA+ICSI, and PGT (PGT-A). Their reasoning part was carefully crafted and covered all critical steps. The complete prompt is provided in Multimedia Appendix 2. For every test case, this same curated set of 6 diverse examples was prepended to the prompt. The purpose of this strategy was to provide the model with comprehensive and representative clinical guidance within the prompt itself.

In summary, this 3-arm design allows for a multifaceted analysis of CoT reliability. The comparison between group 1 and group 2 will isolate the general benefit of using in-context examples. The critical comparison between group 2 and group 3 will determine whether our proposed selective prompting strategy provides a statistically significant improvement over a random baseline. Together, these comparisons will build a clear evidence-based argument for the importance of a well-designed prompting strategy in generating reliable and accurate clinical reasoning.

### Evaluation Metrics

#### Physician Evaluation

The evaluation was conducted by a panel of 2 board-certified reproductive physicians. Both subject matter experts are faculty members at the same academic medical center but work independently in separate clinical teams. They were invited through an internal clinical research collaboration mechanism, and participation was voluntary. Their sole role was to perform a blinded evaluation of the CoTs. Each evaluator possesses over 10 years of clinical experience in the field of ART. Prior to the formal evaluation, a calibration session was held where all evaluators scored 10 cases together. Any discrepancies were discussed to ensure a consistent understanding of the criteria. During the blinded evaluation, each physician reviewer received only the clinical vignette and the model-generated reasoning content. All identifying information, model names, and prompting strategies were removed to ensure full masking. The order of cases and strategies was independently randomized for each reviewer to prevent any presentation bias. Each physician independently scored the complete dataset without discussing their ratings with other reviewers. No reviewer saw any ground-truth labels or model metadata during the evaluation process.

In this study, we created an evaluation metric involving 3 dimensions: logical coherence and clarity (LCC), use and coverage of key information (UCKI), and plausibility and clinical accuracy of reasoning (PCAR). All generated CoTs were scored by the 5-point Likert scale (1=poor and 5=excellent) across 3 key dimensions of reliability, as shown in Table 2 and detailed in Multimedia Appendix 3. All paired comparison results are tested using the Wilcoxon test and adjusted for false discovery rate (FDR).

**Table 2:** Rubric for the evaluation of chain-of-thought (CoT) reliability^a^.

Metric	Definition
Logical coherence and clarity	Assesses whether the reasoning process is internally consistent, logically structured, and expressed clearly and understandably.
Use and coverage of key information	Evaluates the extent to which the reasoning incorporates and addresses relevant clinical data points presented in the input.
Plausibility and clinical accuracy of reasoning	Measures whether the reasoning is clinically sound, aligns with standard medical knowledge, and leads to a reasonable interpretation or decision. Deduct points as appropriate across the 4 parts in the analysis.

^a^The table defines the 3 dimensions: logical coherence and clarity, use and coverage of key information, and plausibility and clinical accuracy of reasoning, used by both human experts and the artificial intelligence evaluator to assess the quality of generated CoTs on a 5-point Likert scale.

#### AI Grader Evaluation

In addition to manual evaluation conducted by human experts, we implemented a supplementary evaluation component leveraging a widely used LLM verifier [35], GPT-4o, to explore its feasibility as an automated evaluator of clinical reasoning. This design enables a direct comparison between AI-generated assessments and the human gold standard, thereby evaluating the feasibility of using LLMs for quality control in large-scale synthetic dataset generation. To ensure comparability, the evaluation criteria provided to the AI model were identical to those outlined in Table 2, including definitions of logical coherence, clinical appropriateness, and key information use. The detailed prompt can be found in Multimedia Appendix 2. Each instruction consists of 2 blocks: the evaluation rubric and case vignette.

### Statistical Analysis

All statistical analyses were conducted in Python (pandas, SciPy, and statsmodels; Python Software Foundation). Each case (N=200) was independently evaluated under 3 prompting strategies (zero-shot, random few-shot, and selective few-shot) across 3 dimensions: LCC, UCKI, and PCAR. Results are reported as mean (SD), with exact n values indicated in tables. To assess the reliability of the physician ratings, we evaluated agreement using three complementary measures: (1) linear weighted κ, (2) adjacent agreement rate (percentage of paired scores within ±1 point), and (3) raw interrater disagreement rates. Because the rating distributions exhibited strong ceiling effects (scores concentrated around 4-5), linear weighted κ is known to underestimate agreement under low variance conditions, a well-described statistical paradox. Therefore, in addition to reporting κ values for completeness, we emphasized adjacent agreement and disagreement rates, which more accurately capture clinical consensus when rating scales are narrow. Across all 600 evaluations (200 cases×3 strategies), disagreement rates were uniformly low, confirming strong consistency between the 2 raters. The selective few-shot strategy showed the lowest disagreement (LCC: 0.00, PCAR: 0.03, UCKI: 0.02), followed by the zero-shot (LCC: 0.04, PCAR: 0.04, UCKI: 0.07) and random few-shot strategies (LCC: 0.12, PCAR: 0.09, UCKI: 0.07). The highest disagreement observed across all metrics was only 12%. Adjacent agreement was correspondingly high, ranging from 88% to 100% depending on the metric and strategy, indicating excellent practical concordance despite the compressed scoring range. Given these properties, interrater reliability was interpreted primarily through adjacent agreement and disagreement rates, while κ statistics were retained as a formal but secondary indicator.

Normality of paired differences was assessed using the Shapiro-Wilk test and Q-Q plots. Because the evaluation scores are 5-point Likert ratings, the paired differences take on only a small number of discrete values (–2, –1, 0, +1, +2). As expected with large samples and discrete Likert data, the Shapiro-Wilk was extremely sensitive and returned significant results; however, Q-Q plots showed no meaningful deviations from approximate linearity beyond the expected discreteness. Parametric tests are generally robust to moderate deviations from normality in Likert-type data, especially with larger sample sizes. We retained the nonparametric Wilcoxon paired tests as primary analyses. All the statistical test details can be checked in Multimedia Appendix 4.

To account for multiple pairwise comparisons across the prompting conditions and evaluation metrics in the subgroups, adjusted *P* values were calculated using the Benjamini-Hochberg FDR correction across all 9 contrasts (3 comparisons per metric). For each metric, paired comparisons were conducted using Wilcoxon paired tests, and both the raw and FDR-adjusted *P* values were reported. A post-hoc power analysis was performed. For each evaluation metric, Cohen *d* effect sizes were computed using the pooled SD of paired observations. This analysis was conducted to assess the stability of estimates under small-sample conditions.

## Results

### Overview

All the results were obtained through the evaluation dataset (N=200), including several kinds of ART. As mentioned earlier, 3 metrics were used for evaluation: LCC, UCKI, and PCAR. The evaluation was done by a panel of experienced practitioners.

### General Performance

Table 3 presents the average scores of each prompting strategy on LCC, UCKI, and PCAR. The selective few-shot strategy outperformed both zero-shot and random few-shot approaches across all 3 metrics. Specifically, it achieved mean scores of 4.56 (SD 0.50), 4.66 (SD 0.53), and 4.18 (SD 0.56), which were significantly higher than those of the zero-shot strategy (mean 4.18, SD 0.56; mean 4.30, SD 0.63; mean 3.85, SD 0.53, respectively; all *P* and adjusted *P*<.001; Cohen *d*=0.72, 0.61, 0.61) and the random few-shot strategy (mean 4.31, SD 0.64; mean 4.42, SD 0.58; mean 3.91, SD 0.63, respectively; all *P* and adjusted *P*<.001; Cohen *d*=0.45, 0.42, 0.46).

**Table 3:** The performance of the “zero-shot,” “random few-shot,” and “selective few-shot” strategies (N=200 cases per group)^a^.

Strategy	LCC^b^, mean (SD)	UCKI^c^, mean (SD)	PCAR^d^, mean (SD)
Zero-shot	4.18 (0.56)	4.30 (0.63)	3.85 (0.53)
Random few-shot	4.31 (0.64)	4.42 (0.58)	3.91 (0.63)
Selective few-shot	4.56 (0.50)	4.66 (0.53)	4.18 (0.56)

^a^Wilcoxon test used for paired comparisons; *P* values adjusted using the Benjamini-Hochberg false discovery rate procedure.

^b^LCC: logical coherence and clarity.

^c^UCKI: use and coverage of key information.

^d^PCAR: plausibility and clinical accuracy of reasoning.

Notably, there was no statistically significant difference between the zero-shot and random few-shot groups on PCAR, though samples did improve the model’s capability on LCC and UCKI statistically significantly.

### Subgroup Analysis

To further dig into the reasons for selective few-shot’s winning, we did an analysis grouped by ART. Table 4 presents the scores of 3 ART generations.

**Table 4:** Subgroup analysis by assisted reproductive technology (ART) category^a^.

ART and strategy		LCC^b^	UCKI^c^	PCAR^d^
**IVF^e^**				
	Zero	4.20 (0.57)	4.34 (0.61)	3.88 (0.53)
	Random	4.29 (0.67)	4.44 (0.53)	3.90 (0.65)
	Selective	4.59 (0.49)	4.69 (0.51)	4.20 (0.55)
**ICSI^f^**				
	Zero	4.16 (0.59)	4.29 (0.65)	3.87 (0.53)
	Random	4.37 (0.54)	4.45 (0.69)	3.97 (0.59)
	Selective	4.45 (0.50)	4.53 (0.60)	4.11 (0.56)
**PGT^g^**				
	Zero	4.09 (0.43)	4.05 (0.72)	3.64 (0.49)
	Random	4.32 (0.57)	4.27 (0.70)	3.86 (0.56)
	Selective	4.59 (0.50)	4.68 (0.48)	4.18 (0.59)

^a^Subgroup analyses were based on the respective case counts (IVF: n=140; ICSI: n=38; PGT: n=22). This table aims to further investigate the performance differences of various prompting strategies across specific clinical scenarios. To achieve this, we categorized the 200 evaluation cases based on their primary type of ART, including IVF, ICSI, and PGT, and conducted a comparative analysis of evaluation outcomes within each group. Wilcoxon test used for paired comparisons; *P* values adjusted using the Benjamini-Hochberg false discovery rate procedure.

^b^LCC: logical coherence and clarity.

^c^UCKI: use and coverage of key information.

^d^PCAR: plausibility and clinical accuracy of reasoning.

^e^IVF: in vitro fertilization.

^f^ICSI: intracytoplasmic sperm injection.

^g^PGT: preimplantation genetic testing.

In the largest subgroup, IVF (n=140), a key distinction emerged. While the selective few-shot strategy significantly outperformed both other groups across all metrics (*P*<.001 and adjusted *P*<.001 for all comparisons, selective vs zero: Cohen *d*=0.72, 0.61, 0.59; selective vs random: Cohen *d*=0.51, 0.49, 0.50), there was no statistically significant difference observed between the random few-shot and zero-shot strategies (*P*=.19, .69, .10; adjusted *P*=.22, .69, .13).

The analysis of the PGT subgroup (n=22) revealed the clearest advantage for prompt diversity. The selective few-shot strategy, which was the only prompt containing a PGT example, outperformed the random few-shot strategy across all 3 metrics: logical coherence (LCC: *P*=.03; adjusted *P*=.05; Cohen *d*=0.51), information use (UCKI: *P*<.001; adjusted *P*=.01; Cohen *d*=0.55), and clinical accuracy (PCAR: *P*=.03; adjusted *P*=.05; Cohen *d*=0.68). Consistent with other findings, the random few-shot strategy showed no significant improvement over the zero-shot baseline in this category (LCC: *P*=.17; adjusted *P*=.19; Cohen *d*=0.45; UCKI: *P*=.27; adjusted *P*=.27; Cohen *d*=0.32; PCAR: *P*=.13; adjusted *P*=.17; Cohen *d*=0.43, respectively). However, we must admit that the limited sample size makes the subgroup analysis partially underpowered, and these comparisons should be interpreted with caution.

A similar pattern emerged in the ICSI subgroup (n=38). The selective few-shot strategy again demonstrated a measurable advantage. It achieved statistically significant improvements over the zero-shot baseline in 2 of the 3 key metrics: LCC (*P*=.02; adjusted *P*=.15; Cohen *d*=0.53) and PCAR (*P*=.04; adjusted *P*=.17; Cohen *d*=0.44). Although these comparisons did not remain significant after FDR adjustment, the effect sizes were moderate, and the directionality was consistent with the overall findings. For UCKI, the selective strategy again achieved the highest mean score, but this comparison did not reach significance (*P*=.06; adjusted *P*=.17), suggesting a positive but statistically inconclusive trend. The detailed subgroup statistical results can be checked in Multimedia Appendix 4.

### Case Study

**Figure 2:**
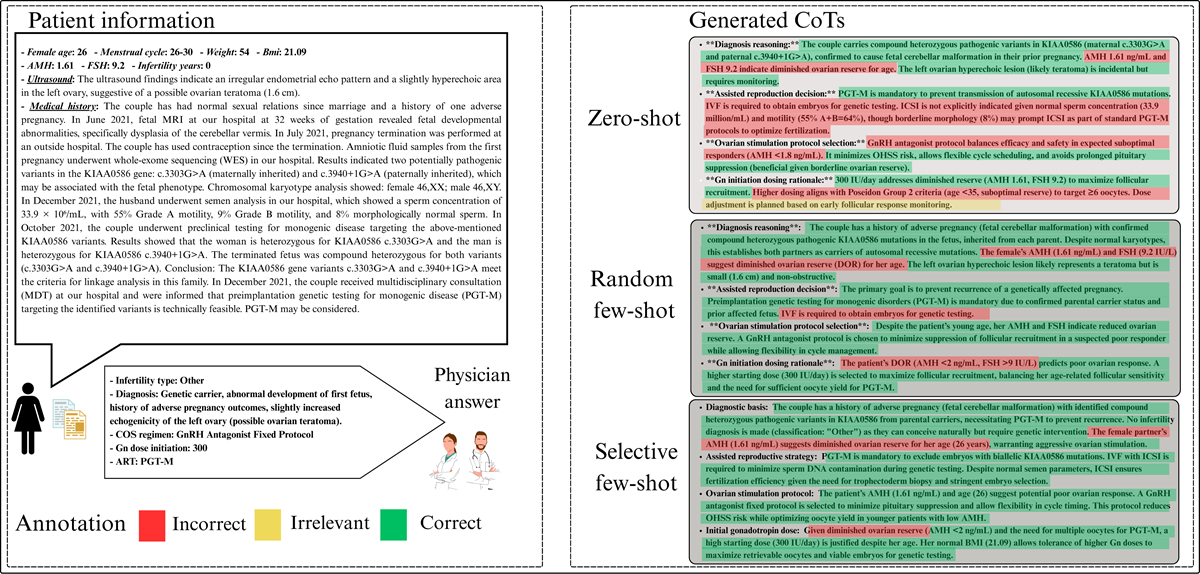
Representative PGT-M case illustrating qualitative differences in CoT reasoning across prompting strategies. This figure presents a representative and complex case involving PGT-M, selected to qualitatively illustrate the differences in reasoning quality observed in our quantitative analyses. The left panel shows the patient’s clinical information, the correct physician’s answer, and the color-coded annotation scheme (red: incorrect reasoning, yellow: irrelevant reasoning, and green: correct reasoning). The right panel displays the CoT outputs generated under zero-shot, random few-shot, and selective few-shot prompting strategies. Compared with the zero-shot and random few-shot generations, which omitted critical reasoning steps (eg, the presence of infertility diagnosis, the indication for intracytoplasmic sperm injection, and comprehensive gonadotropin dose considerations), the selective few-shot prompting was more closely aligned with clinical logic and included relevant patient-specific factors. CoT: chain-of-thought; PGT-M: preimplantation genetic testing for monogenic disorder.

As shown in Figure 2, to qualitatively illustrate the stark differences in reasoning quality revealed by our quantitative analysis, we selected a representative and complex case involving PGT-M. This case is particularly illustrative, as it requires a multilayered understanding of genetics, ART procedures, and individualized patient factors. The main mistakes are listed in Table 5.

**Table 5:** Common reasoning errors in zero-shot and random few-shot chain-of-thought (CoT) outputs for a preimplantation genetic testing for monogenic disorder case.

Reasoning dimension	Flaws in zero-shot and random few-shot
Diagnosis reasoning	The model does not mention whether the patient has infertility issues.
Assisted reproduction decision	CoT incorrectly assumes that if the male’s semen is normal, traditional IVF^a^ can be used.
Ovarian stimulation protocol selection	The reason for choosing the antagonist protocol in CoT was “greater safety and avoidance of OHSS^b^,” without considering the patient’s specific circumstances (low AMH, first ovulation induction).
Initial gonadotropin dose	Only AMH^c^ levels were considered, without taking into account weight, BMI, or PGT^d^ goals (requiring more embryos).

^a^IVF: in vitro fertilization.

^b^OHSS: ovarian hyperstimulation syndrome.

^c^AMH: anti-Müllerian hormone.

^d^PGT: preimplantation genetic testing.

In this PGT-M case, both partners are carriers of a pathogenic variant in the KIAA0586 gene. During a previous pregnancy, the fetus was found to have a homozygous mutation in KIAA0586, resulting in abnormal brain development and subsequent pregnancy termination. Since then, the couple has been using contraception and therefore does not meet the criteria for an infertility diagnosis. This implies that they are still capable of conceiving naturally. Given the autosomal recessive inheritance pattern, there remains a possibility of achieving a normal or carrier embryo through natural conception. However, neither the zero-shot nor the random few-shot–prompted CoT generations mentioned the presence or absence of an infertility diagnosis, which appeared in the selective few-shot–prompted CoT.

To avoid the recurrence of a fetus with a homozygous mutation in KIAA0586, PGT-M is recommended. Due to the technical requirements of PGT, embryos must be obtained via ICSI to avoid DNA contamination during genetic analysis. While the zero-shot and random few-shot–prompted CoTs correctly reasoned the indication for PGT-M, they incorrectly concluded that ICSI was unnecessary because the male partner had normal semen parameters and suggested using conventional IVF instead—an error in clinical reasoning.

In selecting the ovarian stimulation protocol, clinical reasoning typically begins with evaluating the patient’s ovarian responsiveness and any prior stimulation history. Although the patient is 26 years of age, her AMH level is only 1.61 ng/mL, suggesting a potential for diminished ovarian response. As this is her first controlled ovarian hyperstimulation cycle, a gonadotropin-releasing hormone antagonist protocol was chosen for its controllability and to avoid excessive pituitary suppression. Among the 2 few-shot–prompted CoTs, the reasoning was more aligned with clinical thinking, while the zero-shot CoT emphasized the safety profile of the antagonist protocol (eg, avoiding ovarian hyperstimulation syndrome) without clearly reflecting clinical logic.

Regarding the initial gonadotropin dose, factors beyond ovarian responsiveness must be considered. Since this case involves PGT, it is important to optimize the number of oocytes retrieved. Additional considerations include the patient’s weight and BMI, as these affect drug sensitivity. However, the zero-shot CoT mentioned only ovarian responsiveness, lacking a comprehensive rationale.

### Feasibility Analysis of an AI Evaluator

As detailed in Table 6, the mean scores for all 3 prompting strategies were tightly clustered in a narrow and high-scoring range, between 3.96 and 4.00, suggesting that the model perceived all generated outputs as being of similarly high quality.

**Table 6:** Artificial intelligence (AI)–driven evaluation of chain-of-thought reliability across different prompting strategies^a^.

Group	LCC^b^, mean (SD)	PCAR^c^, mean (SD)	UCKI^d^, mean (SD)
Random few-shot	4.00 (0.00)	3.98 (0.14)	4.00 (0.00)
Selective few-shot	4.00 (0.00)	3.98 (0.16)	4.00 (0.07)
Zero-shot	4.00 (0.07)	3.96 (0.20)	3.98 (0.14)

^a^The high scores and minimal variation across all groups indicate a significant ceiling effect in the AI’s evaluation. Paired comparisons between strategies were conducted using the Wilcoxon signed rank test; *P* values were adjusted using the Benjamini-Hochberg false discovery rate procedure.

^b^LCC: logical coherence and clarity.

^c^UCKI: use and coverage of key information.

^d^PCAR: plausibility and clinical accuracy of reasoning.

Inferential statistical analysis corroborated this observation. A series of Friedman tests found no statistically significant differences among the 3 groups for LCC (*P*=.37), PCAR (*P*=.37), or UCKI (*P*=.07). While a post-hoc pairwise Wilcoxon paired test identified a marginal statistical difference between the random few-shot and zero-shot groups on the information use dimension (*P*=.045), this isolated finding merits cautious interpretation, particularly, as the overall test for this dimension did not reach statistical significance.

## Discussion

### Principal Findings

This study critically evaluates the reliability of LLM-generated CoT reasoning in ART and shows that noncurated prompting methods are insufficient for clinical use. Both zero-shot and random few-shot strategies frequently produced reasoning errors, and random shallow examples offered no meaningful improvement over providing no examples at all. In contrast, the selective few-shot strategy, which is built on the principles of representative diversity and gold-standard depth, substantially improved coherence, information use, and clinical accuracy. These reliability gaps, as well as the strengths of the selective approach, were identifiable only through expert review; automated AI evaluators failed to detect these differences. Together, these findings outline a practical framework for evaluating ART reasoning quality and a feasible pathway for generating trustworthy synthetic clinical data.

The principle of representative diversity was clearly demonstrated in the PGT and ICSI subgroups. The findings provide empirical support for our initial hypothesis. The PGT category shows significantly higher scores, prompted by the selective few-shot approach, which includes an example of PGT-A treatment. The case study also shows errors in understanding and judgment in doctors’ viewing, where zero-shot or random few-shot are more likely to make intrinsic mistakes. Notably, in the ICSI category, although the intergroup differences did not reach statistical significance when compared to the random few-shot group, we observed the same trend as in the PGT category—selective prompting consistently achieved the highest average scores and was significantly higher than zero-shot prompting, which had no difference with the random one. The analyses of both subgroups collectively suggest that a demonstration set covering key procedural subtypes within the domain is essential for enabling the model to evolve from a “specialist” to a “generalist.”

Simultaneously, the principle of gold-standard depth was illustrated in the IVF subgroup. In our main results, we show that the quality of examples may influence the quality of generation. In subgroup analysis, we found that there is no significant difference between the zero-shot prompting and the random few-shot prompting on any subgroup, especially in the IVF subgroup, even if the random arm’s sample cases indeed included 4 standard IVF and 1 short-protocol IVF. It performed ineffective learning under this situation. In this case, the reason may be attributed to the reasoning quality in the prompt. In the experiment design section, we mentioned that the random cases have a relatively concise CoT. This indicates that the LLM exhibits a strong tendency toward pattern imitation when engaging in in-context learning. A low-quality example tends to elicit correspondingly poor reasoning outputs, even if the model has huge potential in text generation. Therefore, this principle emphasizes that each few-shot example must serve as an expert-level exemplar: logically rigorous, richly detailed, and representative of ART strategy reasoning at the highest standard.

### Comparison to Prior Work

Our findings align closely with a well-established principle in the broader AI research community: data quality often outweighs data quantity [36]. Our work provides domain-specific empirical support for the application of this principle in the reproductive medicine context of clinical CoT generation. More importantly, we go beyond simply affirming the importance of data reliability; we offer a concrete characterization of what high-quality examples mean in this setting, through our proposed dual principles of gold-standard depth and representative diversity. Together, these insights contribute a practical methodology for realizing data-centric AI specializing in reproductive medicine.

Another key finding highlights a critical limitation of current LLM-based evaluators in detecting subtle yet clinically meaningful variations in information use, logical rigor, and contextual accuracy. While our human expert assessments revealed substantial differences in reasoning quality across the 3 prompting strategies, the scores assigned by the AI evaluator (GPT-4o) showed no statistically significant differences between them across 3 metrics. This “ceiling effect” serves as a critical warning: in high-stakes medical applications, like ART strategy choosing, where patient safety is on the line, relying solely on automated evaluation for quality assurance is inherently risky. It reaffirms that domain expert oversight is not merely a “gold standard” for evaluation; it is an essential safeguard that cannot be replaced. Our results show that AI-based evaluation cannot be treated as a source of ground truth; all judgments involving factual accuracy, clinical appropriateness, or safety must rely on human experts. From a broader methodological perspective, the results underscore a growing challenge for the field. As the development of medical LLMs increasingly depends on large-scale synthetic data, evaluation may become the primary bottleneck. While models continue to improve in producing fluent clinical narratives, reliably detecting subtle but clinically meaningful reasoning errors remains far more difficult. Without dependable evaluators, synthetic or augmented clinical data cannot safely be incorporated into model training pipelines. Addressing this gap will require medically grounded evaluation frameworks, including domain-specific supervision signals, error-aware reward models, and structured representations of clinical logic. These capabilities are not yet captured by current general-purpose LLM judges, emphasizing the need for future research focused on building evaluators that meet the safety, sensitivity, and domain expertise required for clinical AI applications.

This study provides evidence within a single-center ART dataset, and further multicenter generalization is needed. Although we attempted to determine the reliability of AI-generated CoT in complex clinical cases, our cases are currently limited to reproductive medicine or ART treatment. To enhance generalizability and robustness, future research should include a more diverse set of complex clinical reasoning cases across different medical departments. This study has several limitations. First, all generations were produced using a single model (DeepSeek-R1), which restricts the external validity of the findings. Future studies will evaluate whether the advantages of the selective few-shot strategy generalize across different LLM families. Second, the use of temperature=0.5 introduces controlled stochasticity into the inference process; other decoding settings may produce output variations. To address this, future work will include sensitivity analyses across multiple temperature levels (eg, 0, 0.2, and 0.5) to assess the stability of reasoning patterns. In addition, all human evaluators were recruited from the same medical center, which may introduce institutional bias due to shared training backgrounds and practice standards. The AI-grader feasibility test also has limitations: the grader’s sensitivity is partly dependent on its prompt design and model chosen, which may reduce its ability to detect subtle but clinically important differences within the reasoning block. Finally, the evaluation was conducted at the case level, and although the Likert-based rubric captures overall reasoning quality, subjective variability cannot be fully eliminated. Future work may incorporate sentence-level or error-type–specific analysis to support more objective and fine-grained identification of reasoning deficiencies. Given these constraints, this study should be interpreted as a vertical, domain-specific proof-of-concept, rather than a horizontal benchmark applicable across clinical specialties or model families. The selective few-shot strategy was examined within ART because it provides a well-defined and clinically coherent setting for studying structured reasoning, not because its performance should be assumed to generalize elsewhere. Whether the observed improvements reflect a domain-specific phenomenon or a more general pattern cannot be determined from this study. Future work will therefore focus on rigorously evaluating the approach across diverse clinical domains, datasets, and model families to assess its true generalizability. Beyond the constraints and limitations, an important consideration of this study is that the selective few-shot condition differed from the random condition not only in the conceptual selection principles but also in exemplar characteristics, including number, clinical depth, and ART subtype diversity, which creates a mixed signal. Although the numerical difference between 5-shot and 6-shot prompting is small, it may nonetheless introduce bias. More importantly, exemplar depth and subtype diversity were intentionally incorporated to construct a clinically coherent selective prompt, but these factors inherently covary in our current design. As a result, this study cannot attribute the observed improvements to any single component of the selective strategy nor determine whether the effect arises from exemplar depth, diversity, their interaction, or other uncontrolled influences. The findings should therefore be interpreted as exploratory and hypothesis-generating, rather than evidence of a validated mechanism. To address this joint pattern, future work will implement controlled ablation studies that (1) equalize exemplar number across conditions and (2) independently manipulate exemplar depth (“deep vs shallow”) and subtype diversity (“diverse vs homogeneous”). Such studies will allow rigorous assessment of the independent and combined contributions of these factors to few-shot reasoning performance in clinical LLMs.

Our dataset contains 200 diverse cases, but for some subtypes, the number of cases may be too small for statistical analysis, particularly the PGT subgroup, which only included 22 samples. Although the post-hoc power calculation and *P* value correction were conducted, it still showed moderate effect sizes on part of the comparisons. Accordingly, the subgroup findings should be interpreted as exploratory. A potential methodological improvement for future studies is the use of hierarchical partial-pooling or Bayesian shrinkage models, which may borrow strength across subgroups and produce more stable estimates under low-sample conditions. These models were not adopted in this study because our primary objective was descriptive comparison rather than multilevel estimation, but they represent a promising direction for future research. For future directions, given the limited context window of current LLMs, users may face an inherent trade-off when selecting few-shot exemplars, particularly in domains such as reproductive medicine where clinical presentations exhibit substantial subtype diversity. Balancing breadth and depth in exemplar selection becomes a critical challenge under these constraints. Recent work on dynamic prompting methods has sought to improve the performance-efficiency trade-off in resource-limited or accuracy-constrained settings [37], and incorporating such techniques may further enhance the practicality of selective prompting in clinical applications. In addition, future work will explore retrieval-augmented generation frameworks. Integrating authoritative domain sources (eg, American Society for Reproductive Medicine and European Society of Human Reproduction and Embryology guidelines) has the potential to improve factual grounding, reduce hallucination, and enhance explainability in ART-related clinical reasoning. Comparing closed-book reasoning with retrieval augmented generation augmented reasoning may clarify how access to external evidence shapes LLM decision-making and may improve the reliability of LLM-assisted clinical decision support tools.

### Conclusions

The primary contribution of this study is 2-fold: an exploratory potential evaluation framework for how to evaluate and provide a methodology for a feasible approach and for how to generate trustworthy clinical ART reasoning steps in a single clinic center. First, we investigate a rigorous, domain-grounded framework for evaluating synthetic clinical reasoning within the ART strategy. Amid the rapid growth of AI in health care, we demonstrate that ensuring clinical validity requires moving beyond automated metrics. Our findings expose the critical limitations of SOTA AI evaluators (eg, GPT-4o) in detecting subtle but clinically vital reasoning flaws. This “ceiling effect” serves as a critical warning and highlights the indispensable role of structured, blind expert review as an essential safeguard in reproductive medicine AI development. Second, building on this evaluation framework, we offer a practical solution to the “explainability data bottleneck” in reproductive medicine. Through systematic comparisons, we show that a selective few-shot prompting strategy, which is based on the “dual principles” of gold-standard depth and representative diversity, substantially improves the quality and reliability of generated ART CoTs. This offers a feasible, cost-effective blueprint for generating trustworthy ART synthetic data at scale, without requiring immense annotated datasets. Finally, this study evaluates the clinical reliability of LLM-generated reasoning in the ART context as a step toward addressing data scarcity in explainable, domain-specialized AI development. However, our findings should not be interpreted as evidence that current LLMs are clinically safe or ready for autonomous use. Our evaluation focuses on reasoning quality, not deployment readiness. Establishing clinical go or no-go thresholds will require task-specific, prospective validation studies assessing safety, consistency, patient outcomes, and workflow integration—factors beyond the scope of this work.

## Appendix

Multimedia Appendix 1 Detailed description of variables used in the dataset.

Multimedia Appendix 2 Chain-of-thought generation prompt.

Multimedia Appendix 3 Detailed human evaluation rubric.

Multimedia Appendix 4 Statistical results.

## Abbreviations

AI: artificial intelligence
ART: assisted reproductive technology
CoT: chain-of-thought
EHR: electronic health record
FDR: false discovery rate
HIPAA: Health Insurance Portability and Accountability Act
ICSI: intracytoplasmic sperm injection
IVF: in vitro fertilization
LCC: logical coherence and clarity
LLM: large language model
PCAR: plausibility and clinical accuracy of reasoning
PGT: preimplantation genetic testing
PGT-A: preimplantation genetic testing for aneuploidies
PGT-M: preimplantation genetic testing for monogenic disorder
SOTA: state-of-the-art
TESA: testicular sperm aspiration
UCKI: use and coverage of key information

## Data Availability

The datasets generated or analyzed during this study are available from the corresponding author on reasonable request.
