# Supplementary material for: Reliability of Large Language Model Generated Clinical Reasoning in Assisted Reproductive Technology: Blinded Comparative Evaluation Study
Source: J Med Internet Res. 2026 Jan 8;28:e85206. doi: 10.2196/85206 (PMC12828306; doi:10.2196/85206)
Supplement: Multimedia Appendix 1 [file jmir_v28i1e85206_app1.docx]

**Table S1.** Detailed description of the structured and unstructured variables used in the dataset, categorized into Baseline and Demographics, Present Illness History, and Preliminary Diagnosis and Treatment Plan.

| Category | Variable | Description |
| --- | --- | --- |
| Baseline and Demographics | Female age | Age of the female patient at the time of clinical evaluation |
|  | Menstrual cycle | Length of menstrual cycles |
|  | Body weight | Patient’s weight measured in kilograms |
|  | Body Mass Index (BMI) | Calculated BMI based on height and weight, indicating nutritional status |
|  | Anti-Müllerian Hormone (AMH) level | Serum AMH level indicating ovarian reserve |
|  | Duration of infertility | Time period (in years) the couple has attempted conception without success |
|  | Gynecological ultrasound findings | Imaging observations of uterus, ovaries, and antral follicle count |
|  | Baseline follicle-stimulating hormone (FSH) level | Day 2–5 FSH level indicating baseline ovarian function |
| Present Illness History | Present Illness History | Narrative summary of the patient’s current reproductive and clinical symptoms |
| Preliminary Diagnosis and Treatment Plan | Type of infertility | Classification of infertility as primary or secondary |
|  | Controlled ovarian stimulation (COS) protocol | Planned stimulation protocol (e.g., antagonist, long agonist) |
|  | Initial gonadotropin (Gn) dosage | Starting dose of exogenous gonadotropins used for stimulation |
|  | Preliminary differential diagnosis | Initial clinical hypothesis based on presented data |
|  | Initial ART strategy | Selected assisted reproductive technique (e.g., IVF, ICSI, IUI) |
