# Supplementary material for: Reliability of Large Language Model Generated Clinical Reasoning in Assisted Reproductive Technology: Blinded Comparative Evaluation Study
Source: J Med Internet Res. 2026 Jan 8;28:e85206. doi: 10.2196/85206 (PMC12828306; doi:10.2196/85206)
Supplement: Multimedia Appendix 2 [file jmir_v28i1e85206_app2.docx]

S2.1. Zero-shot

| ***You are a highly experienced reproductive medicine specialist. Your task is to provide a step-by-step reasoning process (Chain-of-Thought) that logically explains how to arrive at a GIVEN final treatment plan based on the provided patient information.*  *Your reasoning must be structured into four distinct parts:*  *1.  **Diagnosis reasoning:** Derive the known diagnosis from the provided patient history and clinical data.*  *2.  **Assisted reproduction decision:** Explain the choice of treatment method (e.g., IVF, ICSI).*  *3.  **Ovarian stimulation protocol selection:** Explain the choice of the COS protocol.*  *4.  **Gn initiation dosing rationale:** Explain the initial gonadotropin dose based on all data given.*  *The reasoning should look like: 1. Diagnosis reasoning... 2. Assisted reproduction... 3...*  *The reasoning should be concise, professional, and directly lead to the provided final plan. Please begin your response directly with the analysis. **Reason Authentically as a doctor, Rather Than Explaining The Output and Please keep the chain of thought concise and to the point.***  *Please provide the reasoning process for the following case:*  *{case_information}* |
| --- |

S2.2. Random Few-shot

| {          "input": {              "Female_age": 34,              "Menstrual_cycle": "28-30 days",              "Weight": 48.0,              "BMI": 18.29,              "AMH": 11.38,              "FSH": 9.3,              "Infertility_years": 1.0,              "Ultrasound": "Uterine fibroid (a hypoechoic mass measuring 2.1 × 1.7 × 1.5 cm detected intramurally at the uterine fundus)",              "medical_history": "The couple has been living together and engaging in normal sexual activity since their marriage. The female partner underwent a termination of pregnancy in 2012 and has not used contraception for the past year without achieving pregnancy. Her menstrual cycle is regular, occurring every 28-30 days and lasting for 7 days. In 2008, she had a laparoscopic excision of bilateral ovarian teratomas at another facility. In 2018, she underwent laparoscopic myomectomy at our hospital and used contraception for 2 years post-surgery. In April 2021, a hysterosalpingogram (HSG) performed at our hospital showed normal size and shape of the uterine cavity. The right fallopian tube was fully visualized with good spillage, while the left fallopian tube was fully visualized with poor spillage. The male partner has no previous history of conception. A semen analysis conducted in December 2020 at another facility showed a sperm concentration of 24×10^6/ml, a progressive motility rate of 50%, and a normal morphology rate of 2%."          },          "cot": """Analysis:      1. Diagnosis reasoning:          The couple has been trying to conceive for over one year with regular unprotected intercourse, and the female partner has a history of prior pregnancy. This fulfills the diagnostic criteria for secondary infertility. A hysterosalpingography (HSG) examination showed poor diffusion of contrast in the left fallopian tube, suggesting tubal adhesion. The patient has a surgical history of bilateral ovarian teratoma enucleation and myomectomy, which leads to a diagnosis of a scarred uterus. Current ultrasound findings indicate an intramural uterine fibroid, confirming the diagnosis of uterine fibroid (intramural). Meanwhile, the male partner’s normal sperm morphology rate is 2%, which is below normal threshold, thus the diagnosis of teratozoospermia is established.  2. Assisted reproduction decision:          Given the couple’s history of infertility with a prior pregnancy and the female’s history of two abdominal surgeries, along with imaging evidence of tubal adhesion, the infertility is likely due to tubal/pelvic factors. The male partner’s semen analysis does not show severe abnormalities; therefore, IVF is considered an appropriate and effective option for assisted reproduction.  3. Ovarian stimulation protocol selection:          The female partner is under 35 years old and has a high AMH level, indicating good ovarian reserve and a potential for high ovarian response. To reduce the risk of ovarian hyperstimulation syndrome (OHSS), a GnRH antagonist protocol is selected.  4. Gn initiation dosing rationale:          Given the female partner’s young age (<35) and high AMH, she is expected to be a high responder. Therefore, the initial gonadotropin (Gn) dose is set at 100–150 IU. Additionally, due to her low body weight and low BMI, a relatively lower dose within this range is chosen to minimize the risk of overstimulation.  """,          "output": {              "Infertility_type": "Primary infertility",              "Initial_diagnosis": "Left fallopian tube adhesion; male partner with teratospermia; history of uterine fibroid resection; male partner is a carrier of alpha-thalassemia gene.",              "COS_regimen": "GnRH Antagonist Fixed Protocol",              "Gn_initiation": 100,              "treatment": "IVF"          }      },      {          "input": {              "Female_age": 37,              "Menstrual_cycle": "25 days",              "Weight": 62.0,              "BMI": 25.48,              "AMH": 0.85,              "FSH": 8.4,              "Infertility_years": 7.0,              "Ultrasound": "Uterus and bilateral adnexa appear normal.",              "medical_history": "The couple has been living together and engaging in normal sexual activity since marriage. They have a history of two pregnancies: one induced abortion in 2008 and one full-term cesarean delivery in 2011 (the child is currently healthy). They have been trying to conceive for the past 7 years without success. The female partner has regular menstrual cycles, occurring every 25 days and lasting 3 days. In December 2020, an AMH test conducted at our hospital indicated a level of 0.85 ng/ml, leading to a diagnosis of diminished ovarian reserve. A semen analysis performed on the male partner in December 2020 showed a sperm concentration of 80.5 million/ml, with 24% classified as grade A, 24% as grade B, and 6.2% of sperm with normal morphology."          },          "cot": """Analysis:      1. Diagnosis reasoning:          The couple has had regular unprotected sexual intercourse for 7 years without conception, and the female partner has a history of prior pregnancy. This meets the criteria for secondary infertility. She also has a history of cesarean section, which supports the diagnosis of a scarred uterus. Her anti-Müllerian hormone (AMH) level is 0.85 ng/mL, which is below the normal range, indicating diminished ovarian reserve.  2. Assisted reproduction decision:          The couple is infertile despite a previous pregnancy history. The female partner is older than 35 years, with evidence of reduced ovarian reserve, while the male partner’s semen parameters are normal. Based on these factors, in vitro fertilization (IVF) is considered a suitable assisted reproductive method.  3. Ovarian stimulation protocol selection:          The female partner is of advanced maternal age with low AMH (though not extremely low, i.e., not <0.01 ng/mL), and her menstrual cycles remain regular. Given this profile and the fact that this is the first cycle of controlled ovarian stimulation (COS), a standard GnRH antagonist protocol is selected.  4. Gn initiation dosing rationale:          Due to the female’s advanced age and low AMH, she is expected to be a low responder. Therefore, a starting dose of 150–300 IU of gonadotropins is recommended. Additionally, since the patient has a higher body weight and a BMI in the overweight range, a relatively higher starting dose within this range is selected to optimize follicular response.  """,          "output": {              "Infertility_type": "Secondary infertility",              "Initial_diagnosis": "Decreased ovarian reserve with uterine scarring.",              "COS_regimen": "GnRH Antagonist Flexible Protocol",              "Gn_initiation": 300,              "treatment": "IVF"          }      },      {          "input": {              "Female_age": 32,              "Menstrual_cycle": "20-30 days",              "Weight": 48.0,              "BMI": 17.42,              "AMH": 7.56,              "FSH": 6.3,              "Infertility_years": 6.0,              "Ultrasound": "Ultrasound findings indicate heterogeneous endometrial echogenicity and suspected polycystic changes in both ovaries.",              "medical_history": "The couple has been living together and engaging in normal sexual intercourse without contraception for 6 years without achieving pregnancy. The female partner has a menstrual cycle lasting 6 days, occurring every 20-30 days. In March 2018, she was diagnosed with polycystic ovary syndrome (PCOS). In May 2019, a hysterosalpingogram (HSG) indicated that both fallopian tubes were fully visualized and patent, with good dispersion; the right fallopian tube was elevated at the fimbrial end. In May 2019, the male partner's semen analysis showed a sperm concentration of 193 million/ml, with 36% motility in grade A, 39% in grade B, and a normal morphology rate of 5%. In December 2019, the couple underwent one artificial insemination (AIH) procedure but did not achieve pregnancy."          },          "cot": """Analysis:  1. Diagnosis reasoning:          The couple has been having regular unprotected intercourse for 6 years without achieving pregnancy, and the female partner has no history of prior pregnancy, which fulfills the criteria for primary infertility. The patient has a previous diagnosis of polycystic ovary syndrome (PCOS), with supporting features including menstrual irregularity, polycystic ovarian morphology on ultrasound, and elevated BMI. These clinical findings are consistent with and support the diagnosis of PCOS.  2. Assisted reproduction decision:          The couple is infertile with no prior history of conception and a long duration of infertility. The male partner’s semen analysis is normal, and the female partner has PCOS. Based on these factors, short-protocol IVF is considered an appropriate method of assisted reproduction.  3. Ovarian stimulation protocol selection:          The female partner is relatively young, has a diagnosis of PCOS, and presents with high AMH levels, indicating a high ovarian reserve. To minimize the risk of ovarian hyperstimulation syndrome (OHSS), a GnRH antagonist protocol is chosen for controlled ovarian stimulation.  4. Gn initiation dosing rationale:          Given the patient’s young age, PCOS diagnosis, and high AMH level, she is expected to be a high responder. Therefore, a starting dose of 100–150 IU of gonadotropins is recommended. Considering her low body weight and BMI indicative of underweight, a lower dose within this range is preferred to reduce the risk of overstimulation.  """,          "output": {              "Infertility_type": "Primary infertility",              "Initial_diagnosis": "Polycystic Ovary Syndrome (PCOS)",              "COS_regimen": "GnRH Antagonist Flexible Protocol",              "Gn_initiation": 125,              "treatment": "Short Protocol IVF"          }      },      {          "input": {              "Female_age": 36,              "Menstrual_cycle": "30-90 days",              "Weight": 51.0,              "BMI": 20.17,              "AMH": 12.32,              "FSH": 6.4,              "Infertility_years": 0.0,              "Ultrasound": "Suspected polycystic changes in the left ovary.",              "medical_history": "The couple has a normal sexual life. The female partner has irregular menstrual cycles, occurring every 30 to 90 days, lasting 3 to 5 days. In 2011, she was diagnosed with polycystic ovary syndrome (PCOS) at an outside facility and has been taking Diane-35 since then, during which time her menstrual cycles have become regular. In 2015, she underwent ovulation induction with letrozole at an outside facility, resulting in a pregnancy that ended in a natural miscarriage at 30+ days of gestation, followed by a dilation and curettage (D&C) procedure. In 2017, a hysterosalpingogram (HSG) revealed left hydrosalpinx. In 2019, she underwent laparoscopic surgery for right tubal pregnancy, which included right salpingectomy and left tubal repair. In 2020, she consulted at our hospital and was considered to have insulin resistance, for which metformin was prescribed. The male partner had a semen analysis in April 2020 at our hospital, showing a sperm concentration of 118.5 million/ml, with 45% motility classified as grade A, 20% as grade B, and 8% normal morphology."          },          "cot": """Analysis:  1. Diagnosis reasoning:          The patient has a prior diagnosis of polycystic ovary syndrome (PCOS), supported by menstrual irregularity, polycystic ovarian morphology on ultrasound, and elevated BMI. She underwent right salpingectomy for ectopic pregnancy and left salpingoplasty for hydrosalpinx, with the final diagnoses including: Chronic salpingitis, Postoperative status after removal of right tubal ectopic pregnancy, Postoperative status after left salpingoplasty for hydrosalpinx. Additionally, the patient has a history of insulin resistance, which further supports the PCOS diagnosis.  2. Assisted reproduction decision:          Although no current diagnosis of infertility is documented, the patient has significant bilateral tubal pathology and history of tubal surgeries, along with PCOS. The male partner’s semen analysis is normal, and the couple has a history of previous pregnancy. Given these findings, IVF is considered an appropriate method of assisted reproduction.  3. Ovarian stimulation protocol selection:          The female partner is of slightly advanced reproductive age, with a diagnosis of PCOS and a very high AMH level, indicating a high ovarian reserve. To reduce the risk of ovarian hyperstimulation syndrome (OHSS), a GnRH antagonist protocol is selected.  4. Gn initiation dosing rationale:          Despite the patient’s slightly older age, the presence of PCOS and very high AMH suggests that she is a high responder. A starting dose of 100–150 IU of gonadotropins is recommended. Given her low body weight and normal BMI, a medium-to-low starting dose within this range is preferred to avoid overstimulation.  """,          "output": {              "Infertility_type": "Other",              "Initial_diagnosis": "Chronic salpingitis, polycystic ovary syndrome, insulin resistance, and Hashimoto's thyroiditis.",              "COS_regimen": "GnRH Antagonist Flexible Protocol",              "Gn_initiation": 125,              "treatment": "IVF"          }      },      {          "input": {              "Female_age": 43,              "Menstrual_cycle": "26-28 days",              "Weight": 52.5,              "BMI": 21.57,              "AMH": 1.11,              "FSH": 5.1,              "Infertility_years": 6.0,              "Ultrasound": "Uterine adenomyosis, weak hypoechoic area in the myometrium of the posterior uterine wall, and a cystic lesion in the left ovary.",              "medical_history": "The couple has been living together and engaging in normal sexual activity without contraception for 6 years, but they have not achieved pregnancy. The female partner has a history of irregular menstrual cycles, occurring every 13-14 days within a 26-28 day range, and has not received specific treatment for this condition. In 2020, she was diagnosed with adenomyosis during an outpatient visit at our hospital and has been under observation since then. In June 2021, her Anti-Müllerian Hormone (AMH) level was recorded at 1.11 ng/ml. The male partner has a previous history of fertility, and a semen analysis conducted in June 2021 showed a sperm concentration of 195 × 10^6/ml, a progressive motility rate of 69%, and a normal morphology rate of 6.5%."          },          "cot": """Analysis:  1. Diagnosis reasoning:          The couple has had regular unprotected intercourse for 6 years without achieving pregnancy, and no history of prior conception, meeting the diagnostic criteria for primary infertility. Ultrasound examination reveals adenomyosis with concomitant uterine fibroids, leading to a diagnosis of adenomyosis combined with uterine fibroids. The patient is of advanced maternal age, and her AMH level is 1.11 ng/mL, indicating diminished ovarian reserve. Menstrual history shows menstrual periods lasting 13–14 days with a cycle length of 26–28 days, suggestive of menstrual irregularity, likely secondary to reduced ovarian reserve.  2. Assisted reproduction decision:          The couple is infertile with no prior pregnancy history and a long duration of infertility. The male partner’s semen analysis is normal. Short-protocol IVF is a viable option; however, due to the female partner’s advanced age and poor ovarian reserve, oocyte quality may be compromised. Use of rescue ICSI (after IVF failure) may increase the risk of oocyte damage and compromise embryo development. Therefore, it is reasonable to consider proceeding with IVF initially, and if fertilization fails, switch to ICSI in the next cycle.  3. Ovarian stimulation protocol selection:          The female partner is of advanced age and has a low AMH level. As this is her first controlled ovarian stimulation (COS) cycle, a GnRH antagonist protocol is suggested.  4. Gn initiation dosing rationale:          Considering the patient’s age (>40 years), low AMH, and menstrual irregularities, she is highly likely to be a poor ovarian responder. Therefore, a high starting dose of gonadotropins is recommended to improve the chance of adequate follicular recruitment.  """,          "output": {              "Infertility_type": "Primary infertility",              "Initial_diagnosis": "Decreased ovarian reserve, adenomyosis with uterine fibroids, menstrual irregularities, hypothyroidism.",              "COS_regimen": "GnRH Antagonist Fixed Protocol",              "Gn_initiation": 300,              "treatment": "IVF"          }      }  ]  def build_cot_generation_prompt(row_data_series, templates=DOCTOR_TEMPLATES):      """      Builds a few-shot prompt for CoT generation from a single row of the DataFrame.      """      prompt = "**You are a highly experienced reproductive medicine specialist. Given the patient information and the actual final treatment plan decided by a human expert, please provide a clear and concise step-by-step reasoning process (Chain-of-Thought) that logically explains how one could arrive at the given treatment plan and diagnosis based on the patient's condition. Your reasoning must reflect expert-level clinical decision-making and strictly follow the given structure. Avoid unnecessary elaboration, and keep the reasoning focused and succinct.**\n\n"      for i, template in enumerate(templates):          prompt += f"--- Example {i+1} ---\n"          prompt += "【Patient Information】\n"          prompt += f"- Age: {template['input']['Female_age']} years\n"          prompt += f"- Menstrual Cycle: {template['input']['Menstrual_cycle']}\n"          prompt += f"- Weight: {template['input']['Weight']} kg\n"          prompt += f"- BMI: {template['input']['BMI']}\n"          prompt += f"- AMH: {template['input']['AMH']} ng/mL\n"          prompt += f"- FSH: {template['input']['FSH']}\n"          prompt += f"- Infertility Duration: {template['input']['Infertility_years']} years\n"          prompt += f"- Ultrasound Findings: {template['input']['Ultrasound']}\n"          prompt += f"- Medical History Summary: {template['input']['medical_history']}\n\n"          prompt += "【Final Treatment Plan Provided】\n"          prompt += f"- Infertility Type: {template['output']['Infertility_type']}\n"          prompt += f"- Initial Diagnosis: {template['output']['Initial_diagnosis']}\n"          prompt += f"- COS Protocol: {template['output']['COS_regimen']}\n"          prompt += f"- Initial Gn Dose: {int(template['output']['Gn_initiation'])} IU/day\n"          prompt += f"- Treatment Method: {template['output']['treatment']}\n\n"          prompt += f"【Reasoning Process to Reach the Provided Plan】\n{template['cot']}\n\n"      prompt += "--- New Case for Reasoning ---\n"      prompt += "Now, please provide a detailed reasoning process (Start with Analysis:...) for the following new patient to reach the GIVEN final treatment plan. **Remember, Please Reason Authentically as a doctor Rather Than Explaining The Output and Please keep the chain of thought concise and to the point.**:\n\n"        prompt += "【Patient Information】\n"      prompt += f"- Age: {row_data_series.get('input_Female_age', 'N/A')} years\n"      prompt += f"- Menstrual Cycle: {row_data_series.get('input_Menstrual_cycle', 'N/A')}\n"      prompt += f"- Weight: {row_data_series.get('input_Weight', 'N/A')} kg\n"      prompt += f"- BMI: {row_data_series.get('input_BMI', 'N/A')}\n"      prompt += f"- AMH: {row_data_series.get('input_AMH', 'N/A')} ng/mL\n"      prompt += f"- FSH: {row_data_series.get('input_FSH', 'N/A')}\n"      prompt += f"- Infertility Duration: {row_data_series.get('input_Infertility_years', 'N/A')} years\n"      prompt += f"- Ultrasound Findings: {row_data_series.get('input_Ultrasound', 'N/A')}\n"      prompt += f"- Medical History Summary: {row_data_series.get('input_medical_history', 'N/A')}\n\n"      prompt += "【Final Treatment Plan Provided】\n"      prompt += f"- Infertility Type: {row_data_series.get('output_Infertility_type', 'N/A')}\n"      prompt += f"- Initial Diagnosis: {row_data_series.get('output_Initial_diagnosis', 'N/A')}\n"      prompt += f"- COS Protocol: {row_data_series.get('output_COS_regimen', 'N/A')}\n"      prompt += f"- Initial Gn Dose: {int(row_data_series.get('output_Gn_initiation', 0))} IU/day\n"      prompt += f"- Treatment Method: {row_data_series.get('output_treatment', 'N/A')}\n\n"      prompt += "【Reasoning Process to Reach the Provided Plan】\n"      return prompt |
| --- |

S2.3. Selective Few-shot

| DOCTOR_TEMPLATES = [      {          "input": {              "Female_age": 32,              "Menstrual_cycle": "20-30 days",              "Weight": 48.0,              "BMI": 17.42,              "AMH": 7.56,              "FSH": 6.3,              "Infertility_years": 6.0,              "Ultrasound": "Homogeneous endometrial echo and Suspicious polycystic changes in both ovaries",              "medical_history": "The couple has been living together and engaging in normal sexual intercourse without contraception for 6 years without achieving pregnancy. The female partner has a menstrual cycle lasting 6 days, occurring every 20-30 days. In March 2018, she was diagnosed with polycystic ovary syndrome (PCOS). In May 2019, a hysterosalpingogram (HSG) indicated that both fallopian tubes were fully visualized and patent, with good dispersion; the right fallopian tube was elevated at the fimbrial end. In May 2019, the male partner's semen analysis showed a sperm concentration of 193 million/ml, with 36% motility in grade A, 39% in grade B, and a normal morphology rate of 5%. In December 2019, the couple underwent one artificial insemination (AIH) procedure but did not achieve pregnancy."          },          "cot": """Analysis:      1. Diagnostic Basis:  The couple has engaged in regular unprotected sexual intercourse for 6 years without conception and has no history of prior pregnancy, consistent with primary infertility. The patient has a previous diagnosis of polycystic ovary syndrome (PCOS), with current menstrual irregularities and ultrasound findings of polycystic ovarian morphology, supporting this diagnosis. Hysterosalpingography (HSG) shows bilateral tubal patency, with no history of pelvic or abdominal surgery, and no genital tract abnormalities on ultrasound, thus pelvic or tubal factors are not currently considered. According to the WHO 6th edition criteria, the male partner’s semen analysis is normal, with no evidence of male-factor infertility.  2. Assisted Reproductive Strategy:  Given the history of infertility, patent fallopian tubes, and normal semen analysis, intrauterine insemination (IUI/AIH) is a feasible first-line option. However, considering the primary infertility, long duration of infertility, and previous AIH failure, fertilization disorders cannot be excluded; thus, Short Protocol IVF is recommended. If fertilization fails, rescue ICSI may be performed.  3. Ovarian Stimulation Protocol:  The female partner is under 35 years old, has PCOS, and elevated AMH, indicating high ovarian response potential. A GnRH antagonist protocol is preferred to reduce the risk of ovarian hyperstimulation syndrome (OHSS).  4. Initial Gonadotropin Dose:  For patients with high ovarian response potential, 100–150 IU of gonadotropins is recommended. Given the patient’s low body weight (48 kg) and underweight BMI (<18), a lower starting dose is selected to minimize the risk of OHSS.      """,          "output": {              "Infertility_type": "Primary infertility",              "Initial_diagnosis": "Polycystic ovary syndrome",              "COS_regimen": "GnRH Antagonist Flexible Protocol",              "Gn_initiation": 125,              "treatment": "Short Protocol IVF"          }      },      {          "input": {              "Female_age": 37,              "Menstrual_cycle": "25 days",              "Weight": 62.0,              "BMI": 25.48,              "AMH": 0.85,              "FSH": 8.4,              "Infertility_years": 7.0,              "Ultrasound": "Uterus and bilateral adnexa appear normal.",              "medical_history": "The couple has been living together and engaging in normal sexual activity since marriage. They have a history of two pregnancies: one induced abortion in 2008 and one full-term cesarean delivery in 2011 (the child is currently healthy). They have been trying to conceive for the past 7 years without success. The female partner has regular menstrual cycles, occurring every 25 days and lasting 3 days. In December 2020, an AMH test conducted at our hospital indicated a level of 0.85 ng/ml, leading to a diagnosis of diminished ovarian reserve. A semen analysis performed on the male partner in December 2020 showed a sperm concentration of 80.5 million/ml, with 24% classified as grade A, 24% as grade B, and 6.2% of sperm with normal morphology."          },          "cot": """Analysis:      1. Diagnostic Basis:  The couple has had regular unprotected intercourse without conception for 7 years. With a history of previous pregnancy, this meets the criteria for secondary infertility. AMH is 0.85 ng/mL with normal FSH, indicating diminished ovarian reserve. Given the female partner’s advanced age (>35 years), assisted reproduction is recommended, and tubal evaluation is not necessary. She has a history of cesarean section, consistent with a scarred uterus. Uterine rupture should be considered during embryo transfer. According to WHO 6th edition criteria, the male partner’s semen analysis is normal, with no evidence of male-factor infertility.  2. Assisted Reproduction Strategy:  Given the prolonged infertility (7 years), female age >35, and diminished ovarian reserve, with patient preference, tubal evaluation is unnecessary, and assisted reproduction is advised. With a history of prior pregnancy and normal semen analysis, IVF is appropriate.  3. Ovarian Stimulation Protocol:  The patient is >35 years old with diminished ovarian reserve, suggesting a likely poor ovarian response. As menstrual cycles are regular and FSH is normal, a GnRH antagonist protocol is suitable for the first COS cycle.  4. Initial Gonadotropin Dose:  For patients with expected poor ovarian response, an initial Gn dose of 150–300 IU is recommended. Given the patient’s body weight (62 kg) and slightly elevated BMI (>25), a higher dose of 300 IU is chosen to maximize oocyte yield.  """,          "output": {              "Infertility_type": "Secondary infertility",              "Initial_diagnosis": "Decreased ovarian reserve with uterine scarring.",              "COS_regimen": "GnRH Antagonist Flexible Protocol",              "Gn_initiation": 300,              "treatment": "IVF"          }      },      {          "input": {              "Female_age": 35,              "Menstrual_cycle": "30 days",              "Weight": 50.0,              "BMI": 19.05,              "AMH": 3.57,              "FSH": 9.4,              "Infertility_years": 2.0,              "Ultrasound": "No special results",              "medical_history": "The couple has been trying to conceive for 2 years without success and has had unprotected intercourse. The patient's menstrual cycle is regular, occurring every 30 days and lasting 6 days. A hysterosalpingography performed in 2019 indicated that both fallopian tubes are patent. The male partner's semen analysis from an outside facility revealed azoospermia. Hormonal evaluation showed decreased testosterone and LH levels in the male partner, with a recommendation for consideration of sperm donation. In 2019, the patient underwent two cycles of assisted reproductive techniques using letrozole for ovulation induction, resulting in ovulation but no pregnancy. In August 2020, the patient underwent an assisted reproductive procedure with donor sperm at our facility using a long protocol, resulting in the retrieval of 2 oocytes and 2 embryos, which were cryopreserved. In September 2020, the patient had a frozen embryo transfer of 2 embryos but did not achieve pregnancy."          },          "cot": """Analysis:  1. Diagnostic Basis:  The couple has had regular unprotected intercourse for 2 years without conception, and no history of previous pregnancy is reported, consistent with primary infertility. Semen analysis shows azoospermia, with decreased testosterone (T) and luteinizing hormone (LH) levels, suggesting azoospermia due to hypogonadism or gonadal dysgenesis. The female partner has regular menstrual cycles, bilateral tubal patency, and normal ovarian reserve, with no identifiable female factor infertility.  2. Assisted Reproductive Strategy:  Due to azoospermia and suspected gonadal dysgenesis in the male partner, the likelihood of successful sperm retrieval is low; donor sperm-assisted reproduction is recommended. Despite tubal patency, the female partner has failed two previous attempts of artificial insemination with donor sperm (AID), raising concern for potential gamete transport dysfunction; thus, IVF with donor sperm is advised. Given that fertilization was normal in the previous IVF cycle using donor sperm, continuing with IVF using donor sperm remains the recommended approach.  3. Ovarian Stimulation Protocol:  The female partner is 35 years old, with normal ovarian reserve (AMH 3.57 ng/mL, FSH <10 IU/L). Either a long GnRH agonist protocol or a GnRH antagonist protocol is acceptable. In the previous stimulation cycle using a long protocol, ovarian response was suboptimal (only 2 oocytes retrieved). Therefore, switching to an antagonist protocol is suggested for this cycle.  4. Initial Gonadotropin Dose:  For patients with normal ovarian response, a starting Gn dose of 150–225 IU is typical. Considering the patient’s advanced reproductive age (35 years) and suboptimal previous ovarian response, a higher starting dose is appropriate. However, given her body weight (50 kg) and low BMI (19.05), a balanced starting dose of 225 IU is chosen to reduce the risk of OHSS.  """,          "output": {              "Infertility_type": "Primary infertility",              "Initial_diagnosis": "Male factor infertility",              "COS_regimen": "GnRH Antagonist Flexible Protocol",              "Gn_initiation": 225,              "treatment": "IVF with Donor Sperm"          }      },      {          "input": {              "Female_age": 29,              "Menstrual_cycle": "24-28 days",              "Weight": 60.0,              "BMI": 24.65,              "AMH": 3.52,              "FSH": 7.4,              "Infertility_years": 5.0,              "Ultrasound": "Uterus and bilateral adnexa show no abnormalities.",              "medical_history": "The couple has been living together and engaging in regular sexual activity without contraception for 5 years without achieving pregnancy. The female partner has a generally regular menstrual cycle, lasting 7-8 days every 24-28 days. She reported an HSG performed at an outside facility in 2021, which indicated that both fallopian tubes are patent (report not available). The male partner has a history of teratospermia, with previous semen analysis conducted at an outside facility leading to medication treatment (details unspecified). A semen analysis performed at our hospital in January 2022 showed a concentration of 33.5 million/ml, a progressive motility of 39%, and a normal morphology rate of 0.5%. Most sperm exhibited abnormal head shapes, with approximately 50% of sperm having an acrosome area smaller than 40% of the head. The sperm DNA fragmentation index (DFI) was measured at 52.8% in January 2022. In 2020, the couple underwent IVF at an outside facility, resulting in the retrieval of 20 oocytes and the development of 1 embryo, which was subsequently cryopreserved. After thawing and transferring the embryo, the couple did not achieve pregnancy."          },          "cot": """Analysis:      1. Diagnostic Basis:  The couple has had regular unprotected intercourse for 5 years without conception and no history of prior pregnancy, consistent with primary infertility. According to WHO 6th edition criteria, the male partner’s normal sperm morphology is 0.5% (<1%), indicating severe teratozoospermia. Additionally, a DNA fragmentation index (DFI) of 52.8% suggests significant sperm DNA damage. The female partner has mostly regular menstrual cycles, bilateral tubal patency, normal AMH levels, and no abnormalities on gynecologic ultrasound, with no identifiable female infertility factors.  2. Assisted Reproductive Strategy:  Despite no female factor infertility, the couple has experienced prolonged infertility (5 years), and the male partner presents with severe teratozoospermia and sperm DNA fragmentation. Therefore, ICSI is recommended. In the previous IVF cycle, 20 oocytes were retrieved, but only 1 embryo was obtained, indicating possible fertilization failure, further supporting the use of ICSI in this cycle.  3. Ovarian Stimulation Protocol:  The female partner is <35 years old, with normal AMH (3.52 ng/mL) and FSH <10 IU/L, indicating normal ovarian response. Either a long GnRH agonist protocol or a GnRH antagonist protocol is appropriate. The long follicular phase protocol may improve follicular synchronization, increase the number of developmentally competent embryos, and enhance endometrial-embryo synchrony, thereby improving the chances of fresh embryo transfer—especially considering the suboptimal embryo outcome in the previous cycle.  4. Initial Gonadotropin Dose:  For patients with normal ovarian response, a starting dose of 150–225 IU is recommended. Given the patient’s body weight (60 kg) and a BMI of 24.65 (high-normal), along with a history of high oocyte yield (20 eggs), a moderate dose of 175 IU is chosen to obtain an adequate number of oocytes while minimizing the risk of OHSS.  """,          "output": {              "Infertility_type": "Primary infertility",              "Initial_diagnosis": "Male factor - teratospermia",              "COS_regimen": "Follicular Phase Long-Acting Protocol",              "Gn_initiation": 175,              "treatment": "ICSI"          }      },      {          "input": {              "Female_age": 31,              "Menstrual_cycle": "30-36 days",              "Weight": 62.0,              "BMI": 23.05,              "AMH": 8.07,              "FSH": 4.9,              "Infertility_years": 1.0,              "Ultrasound": "No special results.",              "medical_history": "The couple has been living together for one year and has been trying to conceive without success despite normal sexual intercourse and no contraception. The patient's menstrual cycles are regular, lasting 6 days with a frequency of 30-36 days. Gynecological ultrasound indicates no significant abnormalities in the uterus and bilateral adnexa. The male partner had a semen analysis in April 2020 at an outside facility showing a sperm concentration of 0*10^6/ml. A follow-up examination at our facility also indicated azoospermia. However, a testicular biopsy performed at our facility revealed the presence of mature sperm."          },          "cot": """Analysis:      1. Diagnostic Basis:  The couple has had regular unprotected sexual intercourse for 1 year without conception and no history of prior pregnancy, consistent with primary infertility. Semen analysis revealed a sperm concentration of 0, indicating azoospermia. Testicular sperm aspiration (TESA) retrieved morphologically normal sperm, suggesting obstructive azoospermia (OA). The female partner has regular menstrual cycles, good ovarian reserve, no abnormalities on gynecologic ultrasound, and tubal status not yet evaluated. No female infertility factors identified at this time.  2. Assisted Reproductive Strategy:  In the case of obstructive azoospermia, sperm can be surgically retrieved. The couple may proceed with TESA to obtain testicular sperm, followed by ICSI for fertilization, or consider donor sperm-assisted reproduction. The female partner has good ovarian reserve and is a suitable candidate for controlled ovarian stimulation and oocyte retrieval. The couple has opted for TESA + ICSI-assisted reproduction.  3. Ovarian Stimulation Protocol:  The female is under 35 years old with a high AMH level (8.07 ng/mL), suggesting a normal to high ovarian response. Her menstrual cycle is regular and ultrasound does not show polycystic ovarian morphology. A GnRH antagonist or long GnRH agonist protocol may be used. The long protocol improves follicular synchronization and allows retrieval of a sufficient number of oocytes to compensate for male factor infertility, but requires close monitoring for OHSS.  4. Initial Gonadotropin Dose:  Given the age <35 years and elevated AMH (8.07 ng/mL), a lower starting dose (100–150 IU) is advisable to reduce the risk of OHSS. Considering her body weight (62 kg) and BMI slightly over 25, and the suppressive effect of the long protocol on endogenous FSH, a compromise dose of 125 IU is selected to ensure adequate oocyte yield while minimizing OHSS risk.  """,          "output": {              "Infertility_type": "Primary infertility",              "Initial_diagnosis": "Male factor infertility",              "COS_regimen": "Follicular Phase Long-Acting Protocol",              "Gn_initiation": 125,              "treatment": "TESA+ICSI"          }      },      {          "input": {              "Female_age": 36,              "Menstrual_cycle": "31 days",              "Weight": 52.5,              "BMI": 19.05,              "AMH": 3.2,              "FSH": 13.4,              "Infertility_years": 0.0,              "Ultrasound": "No special results.",              "medical_history": "The female partner is remarried. The couple has normal sexual intercourse. In June 2020, the patient achieved a natural pregnancy, but experienced a miscarriage at 2 months gestation, with a reported chromosomal abnormality of the embryo being monosomy X (report not available). In October 2020, the patient became pregnant again naturally, but at 8 weeks, there was no fetal heartbeat, leading to a subsequent dilation and curettage. They have been practicing contraception since then. The female has regular menstrual cycles, lasting 5 days every 31 days. She reports a cervical polyp excision in 2007 at an outside facility (report not available) and a hysteroscopic polypectomy for endometrial polyps in 2019 at our hospital (report not available). In June 2020, a hysterosalpingography (HSG) at an outside facility showed patent fallopian tubes bilaterally (report not available). The male partner is a carrier of the alpha-thalassemia gene, while the female partner's thalassemia gene screening showed no abnormalities (report not available). In October 2021, a semen analysis at our hospital indicated a sperm concentration of 22.7 million/ml, with 45% motility classified as grade A, 11% as grade B, and 1.5% of sperm having normal morphology. In October 2021, the genetics department at our hospital recommended that the female partner, who has a history of recurrent miscarriage, consider preimplantation genetic diagnosis due to one miscarriage involving an embryo with monosomy X, and advised consultation with the reproductive medicine department."          },          "cot": """Analysis:  1. Diagnostic Basis:  The couple has experienced two consecutive spontaneous miscarriages, one of which was confirmed to be due to embryonic aneuploidy, meeting the criteria for recurrent pregnancy loss (RPL). The female partner is over 35 years old, consistent with advanced maternal age. The male partner’s semen analysis shows 1.5% normal morphology, which, according to the WHO 6th edition criteria, indicates teratozoospermia. The couple has a history of pregnancy, and since the last miscarriage they have used contraception, so no infertility diagnosis is made (classification: “Other”).  2. Assisted Reproductive Strategy:  Although infertility is not diagnosed, due to advanced maternal age and recurrent miscarriage with prior embryonic aneuploidy, the couple opted for PGT-A to reduce the risk of chromosomal abnormalities. Because PGT-A requires ICSI to prevent contamination of genetic material from surrounding sperm, ICSI will be used to achieve fertilization. ICSI also helps overcome the potential negative impact of the male partner’s teratozoospermia on fertilization and embryo development.  3. Ovarian Stimulation Protocol:  AMH = 3.2 ng/mL indicates normal ovarian reserve, but the patient is 36 years old with FSH >10 IU/L, suggesting a potential for poor ovarian response. As this is the first controlled ovarian stimulation (COS) cycle, a GnRH antagonist protocol is recommended as the standard approach to avoid excessive pituitary suppression while minimizing the risk of OHSS.  4. Initial Gonadotropin Dose:  For patients with normal ovarian response, the typical starting dose is 150–225 IU. Considering the patient’s age (36) and elevated FSH (>10), a slightly higher starting dose is reasonable. However, due to her body weight (52.5 kg) and low BMI (19.05), a compromise dose of 225 IU is selected to ensure sufficient oocyte yield while minimizing OHSS risk.  """,          "output": {              "Infertility_type": "Other",              "Initial_diagnosis": "Recurrent miscarriage, advanced maternal age, male factor-teratospermia, chronic hepatitis B virus infection (high viral load), right breast nodule, left breast cyst, history of cervical surgery.",              "COS_regimen": "GnRH Antagonist Fixed Protocol",              "Gn_initiation": 225,              "treatment": "PGT-A"          }      }  ]  def build_cot_generation_prompt(row_data_series, templates=DOCTOR_TEMPLATES):      """      Builds a few-shot prompt for CoT generation from a single row of the DataFrame.      """      prompt = "**You are a highly experienced reproductive medicine specialist. Given the patient information and the actual final treatment plan decided by a human expert, please provide a clear and concise step-by-step reasoning process (Chain-of-Thought) that logically explains how one could arrive at the given treatment plan and diagnosis based on the patient's condition. Your reasoning must reflect expert-level clinical decision-making and strictly follow the given structure. Avoid unnecessary elaboration, and keep the reasoning focused and succinct.**\n\n"      # Add Few-Shot examples from templates      for i, template in enumerate(templates):          prompt += f"--- Example {i+1} ---\n"          prompt += "【Patient Information】\n"          prompt += f"- Age: {template['input']['Female_age']} years\n"          prompt += f"- Menstrual Cycle: {template['input']['Menstrual_cycle']}\n"          prompt += f"- Weight: {template['input']['Weight']} kg\n"          prompt += f"- BMI: {template['input']['BMI']}\n"          prompt += f"- AMH: {template['input']['AMH']} ng/mL\n"          prompt += f"- FSH: {template['input']['FSH']}\n"          prompt += f"- Infertility Duration: {template['input']['Infertility_years']} years\n"          prompt += f"- Ultrasound Findings: {template['input']['Ultrasound']}\n"          prompt += f"- Medical History Summary: {template['input']['medical_history']}\n\n"          prompt += "【Final Treatment Plan Provided】\n"          prompt += f"- Infertility Type: {template['output']['Infertility_type']}\n"          prompt += f"- Initial Diagnosis: {template['output']['Initial_diagnosis']}\n"          prompt += f"- COS Protocol: {template['output']['COS_regimen']}\n"          prompt += f"- Initial Gn Dose: {int(template['output']['Gn_initiation'])} IU/day\n"          prompt += f"- Treatment Method: {template['output']['treatment']}\n\n"          prompt += f"【Reasoning Process to Reach the Provided Plan】\n{template['cot']}\n\n"      # Add the new patient case for the LLM to process      prompt += "--- New Case for Reasoning ---\n"      prompt += "Now, please provide a detailed reasoning process (Start with Analysis:...) for the following new patient to reach the GIVEN final treatment plan. **Remember, Please Reason Authentically as a doctor Rather Than Explaining The Output and Please keep the chain of thought concise and to the point.**:\n\n"        prompt += "【Patient Information】\n"      prompt += f"- Age: {row_data_series.get('input_Female_age', 'N/A')} years\n"      prompt += f"- Menstrual Cycle: {row_data_series.get('input_Menstrual_cycle', 'N/A')}\n"      prompt += f"- Weight: {row_data_series.get('input_Weight', 'N/A')} kg\n"      prompt += f"- BMI: {row_data_series.get('input_BMI', 'N/A')}\n"      prompt += f"- AMH: {row_data_series.get('input_AMH', 'N/A')} ng/mL\n"      prompt += f"- FSH: {row_data_series.get('input_FSH', 'N/A')}\n"      prompt += f"- Infertility Duration: {row_data_series.get('input_Infertility_years', 'N/A')} years\n"      prompt += f"- Ultrasound Findings: {row_data_series.get('input_Ultrasound', 'N/A')}\n"      prompt += f"- Medical History Summary: {row_data_series.get('input_medical_history', 'N/A')}\n\n"      prompt += "【Final Treatment Plan Provided】\n"      prompt += f"- Infertility Type: {row_data_series.get('output_Infertility_type', 'N/A')}\n"      prompt += f"- Initial Diagnosis: {row_data_series.get('output_Initial_diagnosis', 'N/A')}\n"      prompt += f"- COS Protocol: {row_data_series.get('output_COS_regimen', 'N/A')}\n"      prompt += f"- Initial Gn Dose: {int(row_data_series.get('output_Gn_initiation', 0))} IU/day\n"      prompt += f"- Treatment Method: {row_data_series.get('output_treatment', 'N/A')}\n\n"      prompt += "【Reasoning Process to Reach the Provided Plan】\n"      return prompt |
| --- |
