# Supplementary material for: Reliability of Large Language Model Generated Clinical Reasoning in Assisted Reproductive Technology: Blinded Comparative Evaluation Study
Source: J Med Internet Res. 2026 Jan 8;28:e85206. doi: 10.2196/85206 (PMC12828306; doi:10.2196/85206)
Supplement: Multimedia Appendix 3 [file jmir_v28i1e85206_app3.docx]

| Score | Logicality | Accuracy | Information Utilization |
| --- | --- | --- | --- |
| 1 Point  (Very Poor) | The reasoning process is chaotic; logic is severely fractured or self-contradictory. It is completely impossible to understand the connection from Input to output. | The reasoning bases for all four Target Outputs are inconsistent with clinical reality, irrelevant to the conclusion, or forced. The output has "extremely low clinical value." | Key Input information is completely ignored, or the importance of the information is misinterpreted. |
| 2 Points  (Poor) | The reasoning process contains multiple logical obscurities, with obvious gaps or inconsistencies. It is difficult to comprehend the argumentation path. | Some reasoning bases align with clinical thinking, but the reasoning for critical Target Outputs (e.g., "Assisted Reproductive Method", "Ovarian Stimulation Protocol") is incorrect or tenuous. | Some key information is omitted, or there is a significant deviation in the judgment of the information. |
| 3 Points  (Fair) | The reasoning process is generally coherent, though logic in some links is not sufficiently rigorous or contains minor gaps. It is largely understandable. | The reasoning bases for critical Target Outputs (e.g., "Assisted Reproductive Method", "Ovarian Stimulation Protocol") largely align with clinical reasoning. There are irrelevant or incorrect deductions in other areas. | Most key Inputs are mentioned, but their application to the Target Outputs is insufficiently developed or inaccurate. |
| 4 Points  (Good) | The reasoning process is logically clear, with smooth transitions between steps. The argumentation path is defined and easy to follow. | The reasoning bases for the four Target Outputs are consistent with clinical reasoning. Despite minor flaws, they effectively support the clinical diagnosis and treatment plan. | Key Input information is utilized well, and the connections between them and the Target Outputs are established. |
| 5 Points  (Excellent) | The reasoning process is rigorously logical and tightly knit ("meticulous"). Steps are clear and the argumentation is compelling, perfectly demonstrating the logical path from input to output. | The reasoning bases for all four Target Outputs align perfectly with clinical reasoning, providing an accurate and robust foundation for disease assessment and treatment decision-making. | Relevant key Inputs are utilized precisely and comprehensively. The explanation clearly and profoundly elucidates how this information collectively leads to the Target Output. |
