# Supplementary material for: Reliability of Large Language Model Generated Clinical Reasoning in Assisted Reproductive Technology: Blinded Comparative Evaluation Study
Source: J Med Internet Res. 2026 Jan 8;28:e85206. doi: 10.2196/85206 (PMC12828306; doi:10.2196/85206)
Supplement: Multimedia Appendix 4 [file jmir_v28i1e85206_app4.docx]

Table S1. PGT subgroup comparison adjusted statistical analysis.

| Metrics | Comparison | Cohen's d | Adj. P-value (FDR) | Power |
| --- | --- | --- | --- | --- |
| LCC | zero vs random | -0.45 | **0.192** | **0.53** |
| LCC | zero vs selective | -1.07 | 0.002 | **0.998** |
| LCC | random vs selective | -0.51 | 0.046 | **0.62** |
| PCAR | zero vs random | -0.43 | 0.173 | **0.49** |
| PCAR | zero vs selective | -1.01 | 0.001 | **0.994** |
| PCAR | random vs selective | -0.55 | 0.046 | **0.70** |
| UCKI | zero vs random | -0.32 | 0.261 | **0.30** |
| UCKI | zero vs selective | -1.04 | 0.001 | **0.996** |
| UCKI | random vs selective | -0.68 | 0.009 | **0.86** |

Table S2. ICSI subgroup (n = 38) — Wilcoxon Tests with BH-FDR correction.

| Metric | Comparison | p | p_fdr |
| --- | --- | --- | --- |
| LCC | zero vs random | 0.073638 | 0.165686 |
|  | zero vs selective | 0.016377 | 0.147396 |
|  | random vs selective | 0.466854 | 0.525211 |
| PCAR | zero vs random | 0.345779 | 0.466184 |
|  | zero vs selective | 0.049535 | 0.165686 |
|  | random vs selective | 0.362587 | 0.466184 |
| UCKI | zero vs random | 0.216266 | 0.389278 |
|  | zero vs selective | 0.059832 | 0.165686 |
|  | random vs selective | 0.538541 | 0.538541 |

Table S3. IVF subgroup (n = 140) — Wilcoxon Tests with BH-FDR correction.

| Metric | Comparison | p | p_fdr |
| --- | --- | --- | --- |
| LCC | zero vs random | 0.190705 | 0.214543 |
|  | zero vs selective | 3.63×10⁻⁸ | 3.27×10⁻⁷ |
|  | random vs selective | 3.11×10⁻⁵ | 5.07×10⁻⁵ |
| PCAR | zero vs random | 0.691102 | 0.691102 |
|  | zero vs selective | 4.60×10⁻⁷ | 2.07×10⁻⁶ |
|  | random vs selective | 1.92×10⁻⁵ | 4.32×10⁻⁵ |
| UCKI | zero vs random | 0.100906 | 0.129736 |
|  | zero vs selective | 8.02×10⁻⁷ | 2.41×10⁻⁶ |
|  | random vs selective | 3.38×10⁻⁵ | 5.07×10⁻⁵ |

Table S4. PGT subgroup (n = 22) — Wilcoxon Tests with BH-FDR correction.

| Metric | Comparison | | p | | p_fdr | |  |
| --- | --- | --- | --- | --- | --- | --- | --- |
| **LCC** | | zero vs random | | 0.165518 | | 0.186208 | |
|  |  | zero vs selective | | **0.002282** | | **0.006846** | |
|  |  | random vs selective | | **0.033895** | | 0.052213 | |
| **PCAR** | | zero vs random | | 0.131668 | | 0.169287 | |
|  |  | zero vs selective | | **0.001341** | | **0.006033** | |
|  |  | random vs selective | | **0.034808** | | 0.052213 | |
| **UCKI** | | zero vs random | | 0.265037 | | 0.265037 | |
|  |  | zero vs selective | | **0.000967** | | **0.006033** | |
|  |  | random vs selective | | **0.006656** | | **0.014975** | |
